# The blue road: Provenance study of azurite samples from historical locations through the analysis of minor and trace elements

**DOI:** 10.1016/j.heliyon.2023.e19099

**Published:** 2023-08-11

**Authors:** S. Capriotti, L. Medeghini, Silvano Mignardi, M. Petrelli, Michela Botticelli

**Affiliations:** a Department of Earth Sciences, Sapienza University of Rome, P.le Aldo Moro 5, 00185 Rome, Italy; b Department of Physics and Geology, University of Perugia, Piazza dell’Università, 06123 Perugia, Italy; c Kelvin Centre for Conservation and Cultural Heritage Research, School of Culture and Creative Arts, University of Glasgow, Kelvin Hall, 1445 Argyle Street, Glasgow, G3 8AW, UK

**Keywords:** Azurite, EMPA, LA-ICP-MS, Provenance analysis, Blue pigments

## Abstract

The pigments used by artists since ancient times play an important role in historical, artistic, and cultural investigations. They allow the acquisition of useful information for the study of human and technological development. This research aims at differentiating the various sources of azurite exploited in antiquity, based on the study of minor and trace elements. Azurite is one of the most important blue pigments in art history, widely used during the Middle Age and Renaissance. However, very few studies investigated the provenance of the pigment, so today it is still not possible to clearly identify the sources of azurite exploited in the past. This study is based on the analysis of several samples of azurite belonging to the MUST collection (Museum of Earth Sciences, Sapienza University of Rome, Italy) and coming from different historical localities: UK, Italy, Germany, France, Romania and Slovakia (both representative of the resources within the ancient Kingdom of Hungary), Greece and Russia. The samples were analysed by electron microscopy (EMPA and SEM-EDX) and laser ablation-inductively coupled plasma-mass spectrometry (LA-ICP-MS), with the aim of detecting chemical features that are specific to the different azurite ore deposits.

Among the trace elements analysed, Zn, As, Sn, Ca and Sr prove the most suitable for discriminating the origin of the samples, as well as rare earth elements. In particular, Ce and Eu anomalies are suggested as markers for the German and Hungarian localities.

## 1 Introduction

The analysis of pigments used by artists since ancient times has a key role to define human and technological development, as well as trades and connections between populations [1]. Ancient civilizations have always left their mark through painted images that were obtained using natural pigments available nearby or easily made with basic technological process [2]. The first evidence of the use of a blue pigment is in ancient Egypt. Although Egyptian blue has been the main blue pigment in all the Mediterranean area until the end of the Roman period [3], the earliest is said to be azurite [2,4]: rare examples on its use as a pigment can be found in 4th-dynasty decorations on mummy faces (2613 to 2494 BC), more likely as a result of experimentation in Egyptian art [5]. At that time azurite was mostly used as semi-precious stone, analogously to lapis lazuli [6]. Later examples include the use on painted facades of ancient buildings [3] – *i.e*. the Greek temple of Aphaia at Aegina (6th century BC). The pigment became widespread in European painting during the Middle Ages and continued to be popular throughout the Renaissance and later. It was also the main blue pigment used in the Far East, found in wall paintings dating from the Chinese Sung (from 960 AD) and Ming (from 1368 AD) dynasties, and more recently in paintings of the Japanese Ukiyoe school [7].

Azurite is a basic copper carbonate Cu_3_(CO_3_)_2_(OH)_2_ of secondary origin formed by the alteration of Cu-minerals in an alkaline environment with carbonate solutions [8]. It is commonly present as a *patina* or in the oxidised portions of copper veins [9], in the form of prismatic or stalactite crystals or as a massive and earthy deposit [8,10]. Other secondary minerals, often bearing metallic elements, are likely to occur as mineral impurities in azurite, as oxides, carbonates, phosphates, arsenates and sulphates [11].

As a Cu-bearing mineral, azurite is commonly found in deposits exploited for copper, and for this reason it has been the subject of geological and environmental studies. In a report by the U.S. Geological Survey and Department of Interior [12], azurite from the Chinese provenance of Anhui was found associated with malachite, quartz, mica, barite and hematite. Other accessory minerals were cerium-lanthanum monazite (Ce,La,Nd,Th)PO_4_, gypsum CaSO_4_·2H_2_O, and possibly rutile, TiO_2_. High K and Al contents were interpreted as due to the presence of clays. Metals such as silver, nickel, lead, and zinc were found as minor elements.

Provenance studies play an important role for pigment investigations. The analysis of minor and trace elements allows the characterization of geological material and the discrimination of different sources [13]. According to Gettens and Fitzhugh [7], who provided a first list of azurite sources for artists, the main source of azurite in the West was in the ore deposits in the ‘Kingdom of Hungary’ (largely from present-day Slovakia, Romania and Serbia), at least until the mid-seventeenth century when the country, besieged by the Turks, was forced to break relations with Europe. Other sources mentioned by the authors are Chessy (near Lyon, France), and Sardinia (Italy) [7]. All of them are also modern localities. In a more recent review of the pigment, Eastaugh and co-authors [8] also mentioned Redruth, in Cornwall. According to the authors, since Roman times synonyms have been given to the blue pigment to describe its provenance: *Cyprian blue*, for the important copper mines in Cyprus and *Armenium* for the variety with Persian origin cited by Pliny, who also mentions a Spanish sandy form. The same concept is behind the more recent term *azzurro della magna* [7] – possibly matching *azzuro d'allemagna* or *azzurrum de Alemannia* - which relates the extensive mining in several Germany deposits (Goldberg, Saxony; Schwaz, Tirol; Wallerfangen, Saarland), after Hungarian sources had been cut off [11].

Mineralogical-petrographic analyses of paint samples have shown how azurite is often associated with green particles of malachite [13], usually in the form of intergrowths. Other copper minerals, the most common of which are cuprite (Cu_2_O) and tenorite (CuO), respectively in orange-brown and black particles, can be found with azurite [11]. Less frequent is the observation of bright blue-to-green particles of mixite, a Cu–Bi arsenate with chemical formula BiCu_6_(OH)_6_(AsO_4_)_3_(H_2_O)_3_ [14]. The analyses carried out by Salvadó et al. [15] on 15th century wood paintings from the Crown of Aragon described the usual occurrence of barite particles in azurite layers. In the same year Aru et al. [11] were the first to explore a different approach to the subject, based on mineral samples from European mining locations. Using Raman spectroscopy, they confirmed that frequent accessory minerals are malachite, Cu_2_(CO_3_) (OH)_2_, cuprite, Cu_2_O, hematite, Fe_2_O_3_ and goethite, FeO(OH). Minor associations were said to be cerussite, PbCO_3_, cinnabar, HgS, quartz, SiO_2_, calcite, CaCO_3_, rutile and anatase, TiO_2_, rhodochrosite, MnCO_3_, beudantite, PbFe_3_(AsO_4_)(SO_4_)(OH)_6_, and jarosite, KFe_3_(SO_4_)_2_(OH)_6_.

The occurrence of barite had been reported in blue paint layers on Bohemian mural paintings and explained as a natural impurity in the pigment source [16], along with zinc and copper arsenates. It has been stated that even smithsonite, ZnCO_3_, might be present with azurite [17].

Few heritage science investigations have focused on the provenance of azurite using elemental analysis. Minor aluminium and magnesium have been reported in pure pigment samples [13]. Several impurities in natural azurite pigments on illuminated manuscript leaves have been documented by synchrotron X-ray fluorescence (SR-XRF) [18]. Of these, antimony has been linked to Eastern European deposits [9], while bismuth, arsenic and barium have been said to be typical of the Schwarzwald region, Germany [19].

Most of the previous provenance studies on azurite have been carried out on paintings and manuscripts, *i.e.* on the ‘already-processed’ pigment. This means that published results might give account of the effects of crushing and washing procedures, which are known to remove impurities like cuprite [8], but also of the mixing with other pigments. However, these studies cannot provide a precise match with a specific provenance locality. Research into raw materials with known provenance is still essential to understand whether the associated minerals or elements come from the extraction site or are due to pigments used in mixture with azurite.

The present work is based on the study of azurite specimens, originally coming from historical mining localities and now in the MUST collection (Museum of Earth Sciences, Sapienza University of Rome), with the aim of identifying chemical markers for the discrimination of different pigment sources. The systematic evaluation of the structural and chemical characteristics of azurite represents an innovative approach to the provenance issue, to establish supply and trading routes for pigment production in western Europe.

## 2 Materials and methods

### 2.1 Azurite samples

The investigation was carried out on twelve samples of azurite coming from different locations (Table 1) stored in the collection of the Museum of Earth Sciences (MUST), Sapienza University of Rome, Italy. One or more crystals of azurite were selected under a stereomicroscope for each sample.

**Table 1:** List of studied samples with indication of their provenance and a brief description provided by the MUST.

Museum code	Locality	Description
13240/53	Cornwall, England	In aggregate crystals with little limonite
13466/79bis	Campiglia, Tuscany, Italy	With malachite, limonite, pyrite, galena, etc. Gift from Prof. Uzielli
13210/23	Siegen, Westphalia, Germany	Crystals in the cavity of the compact tetrahedra
13216/29	Chessy, Lyon, France	In aggregated crystals partially transformed into malachite with yellow ochre
13228/41	Chessy, Lyon, France	In large crystals aggregated with little yellow ochre and white substance
13238/51	Chessy, Lyon, France	In crystals aggregated with yellow ochre and fibro-ray malachite
14890/83	Wolwodina, Banat, Moldova^a^	Crystals with limonite (with malachite)
13193/6	Gollnitz, Hungary^b^	Imperfect crystals over mixture of tetrahedrite, quartz, limonite
16696/87	Laurion, Athens, Greece	In crystalline crystals that cover the cavity of the limonite
16695/86	Laurion, Athens, Greece	Crystalline crystals covering the cavity of brown limonite with green crystals of calcite
13247/60	Solotuschinsk, Altai, Siberia	Crystals forming a large geode, Spada collection
13202/15	Wolwodina, Banat, Moldova^a^	With light green malachite on *kupfer pekertz*, with little yellow ochre

a The locality is reported according to the Museum catalogue. Wolwodina might correspond to what is now the Autonomous Province of Vojvodina, Serbia. Alternatively, the Museum locality might stand for Moldova Nouă mine, which belongs to Caraş-Severin County, modern Romania. Over the centuries, both Wolwodina and Moldova Nouă mines have been under the Kingdom of Hungary and mentioned in historical sources as Hungarian supplies [11].

b The locality is reported according to the Museum catalogue. Gollnitz is present day Gelnica, a mining town in the Košice Region of Eastern Slovakia. Gölnicbánya is the Hungarian name, Göllnitz the German name.

### 2.2 Sample preparation

Fragments were placed on the base of a cylindrical mould (2.5 cm in diameter), and made adhesive to immobilise the samples. Epoxy resin (3:1 with hardener) was poured into the mould for embedding. Hardening was ensured by exposure to a warm environment (50–70 °C) for 24 h. Sections were then wet-grinded with silicon carbide papers. They were first polished with finer silicon carbide papers (300–1000 grit) and then a diamond paste (6-1 μm) in ethanol was used for the finishing steps.

For electron microscopy (electron microprobe analysis, EMPA, and scanning electron microscopy with energy dispersive X-ray spectroscopy, SEM-EDX) polished sections were coated by deposition of a graphite layer of about 200 Å after thermal evaporation and under high vacuum, to avoid the formation of deep craters on the sample because of the interaction with the electron beam.

Major and minor elements were investigated by EMPA, while trace element analysis was performed by laser ablation-inductively coupled plasma-mass spectrometry (LA-ICP-MS) on the same sections, allowing the qualitative and quantitative chemical analyses of the samples. For each sample, different points on more than one fragment were analysed. This approach was used to obtain results representative both for edges and bulk of the crystal and to study internal variability. SEM-EDX analysis was used to further characterise the type of impurities linked to the major and minor elements previously identified on each sample.

### 2.3 Electron microscopy

Major and minor elements were first investigated by EMPA, using a Cameca SX-50 consisting of 5 WDS spectrometers, all arranged with a take-off angle of 40°, and 12 crystals (CNR–IGAG, Rome, c/o Department of Earth Sciences, Sapienza University of Rome). The selected operating conditions include an acceleration voltage of 15 kV, beam current of 15 nA and beam size of 10 μm for both core and rim. The peaks and background of the elements were measured with counting times of 20 and 10 s respectively. The standards used for the calibration were augite for Mg (TAP, thallium (acid) phthalate crystal), corundum for Al (TAP), barite for S (PET, pentaerythritol crystal), orthoclase for K (PET), wollastonite for Ca (PET), rhodonite for Mn (LIF, lithium fluoride crystal), chalcopyrite for Cu (LIF), sphalerite for Zn (LIF) and barite for Ba (PET). Matrix corrections were calculated by the PAP method [20] using the software supplied by the Microbeams Services. The analytical error was ∼1% rel. for the major elements, and it increases as their concentration decreases. The detection limits under the specified working conditions range between 0.01 and 0.1 wt%.

Impurities were further characterized – chemically and morphologically – by SEM-EDX, according to the specifications and methodology described in Ref. [21].

### 2.4 LA-ICP-MS

Trace element analysis by LA-ICP-MS was carried out at the Department of Physics and Geology of the University of Perugia, Italy. The instrumentation consists of a Teledyne Photon Machine G2 193 Excimer laser ablation system coupled with a Thermo Fisher Scientific iCAP-Q quadrupole-based ICP-MS. The laser beam diameter was 65 μm, the frequency was 10 Hz, and the energy density at the sample surface was 3.5 J/cm^2^. The NIST SRM610 reference material was used for the calibration, while the reference material used for quality control was USGS BCR2G [22]. CuO data obtained with EMPA were used as an internal standard. Data reduction was performed using Iolite3 [23]. Limits of Detection (LOD) in LA-ICP-MS analyses were estimated in agreement with Howell et al. [24]. LA-ICP-MS data, including detection limits, have been reported in Table S3. Statistical data treatment was carried out using Excel (spider plots, histograms and binary plots) and Origin Pro 8.5 (binary and multivariate statistics) software. Finally, Principal Component Analysis (PCA) was carried out on LA-ICP-MS data. The correlation matrix method was used [1], 5 PCs were extracted and the score plot with loadings was obtained to determine whether a relationship exists among samples from the same locality and which are the key elements for the clustering.

## 3 Results

### 3.1 Major and minor elements

**Fig. 1:**
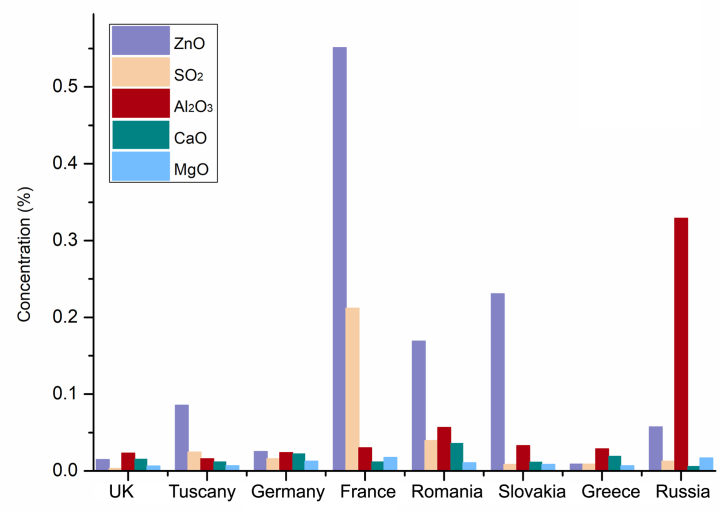
General trend of the average concentrations of the elements investigated through the EMPA analysis in the azurite samples.

From the data obtained by EMPA (Table S1) elevated concentrations of ZnO were found in sample 13216/29 from Chessy (0.4–4.4%). For the same oxide, Gollnitz and Wolwodina showed a significant concentration, with the highest values at 0.86% and 0.5% respectively. SO_2_ content seems to discriminate the French source, while the high content of Al_2_O_3_ characterizes the Siberian sample. The diagram in Fig. 1 shows the average concentrations, calculated from all the measurements within the samples from the same locality, of the oxides investigated through EMPA.

The comparison of the concentrations measured for each point of analysis of the same sample allowed an investigation of the internal variability in composition. For example, the sample from Russia is characterized by high content of Al_2_O_3,_ the highest values (0.68–0.96%) being recorded in the top rim of both the analysed fragments. The French sample 13238/51 showed a high concentration value of SO_2_ in the core (3.01%), which could be considered an outlier. Sulphur is common in azurite because it is linked to its formation and later secondary processes. The oxidation of primary deposits of copper sulphides and/or sulfosalts implies that the sulphur content decreases as a function of the purity of the mineral, leading to the formation of several (lead, zinc) carbonates, and then increases again due to a ‘secondary sulphide enrichment’, i.e. the reaction of copper-rich solutions with primary sulphides to form copper sulphides [25].

**Fig. 2:**
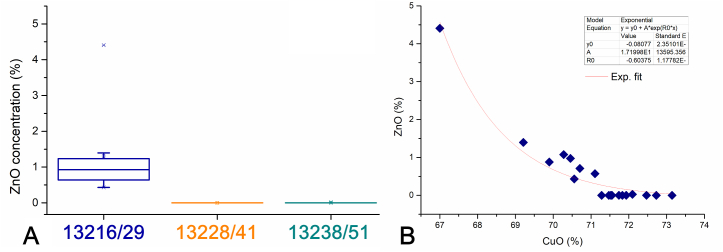
Comparison of EMPA data for the three French samples 13216/29, 13228/41, 13238/51: A) ZnO concentration; B) CuO *vs* ZnO.

ZnO content showed the highest variability within samples from the same locality. For example, it varies remarkably between the three representative samples from Chessy (Fig. 2A). While it is below the detection limit in most of the points of 13228/41 and 13238/51, in 13216/29 the concentration ranges from 0.43 to 1.40%, with one outlier value (4.41%) at the rim.

**Fig. 3:**
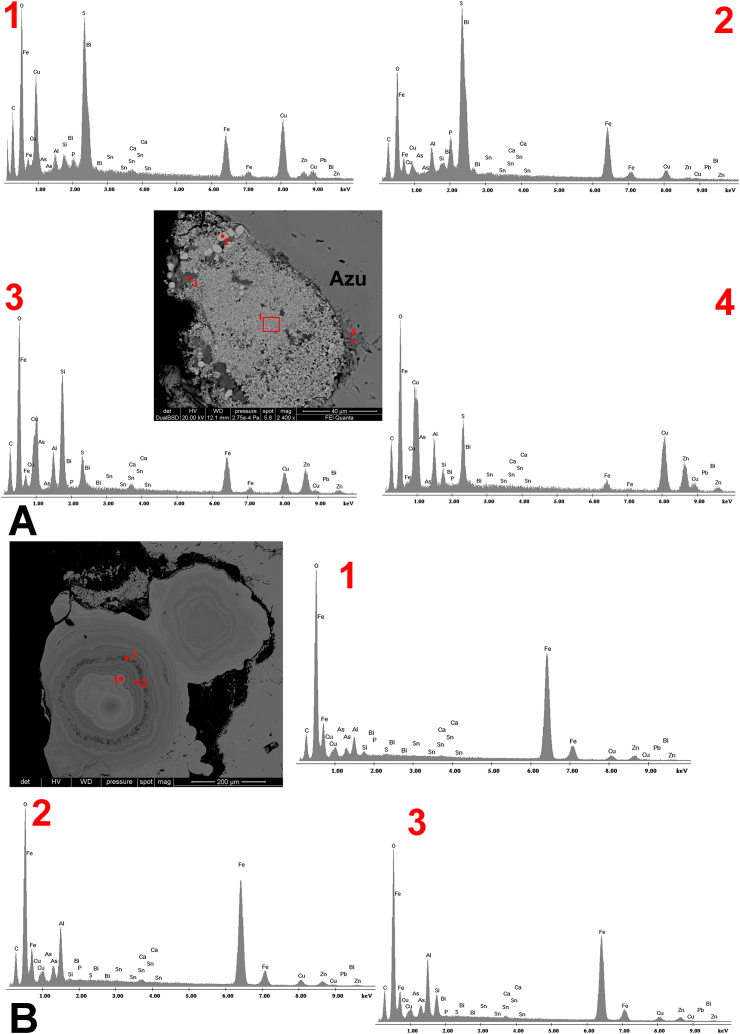
SEM-EDX analysis of different impurities in azurite (labelled ‘Azu’ in each micrograph): A) sample 13216/29 from Chessy, with EDX spectra of (1) chalcopyrite in nanometric spherule, (2) pyrite in micrometric grains, (3) a metal-poor aluminosilicate-rich outer zone with possible deposition of chrysocolla and quartz and (4) an oxidation step between azurite deposition and the secondary sulphide enrichment; B) sample 13193/6 from Slovakia, showing a ferrous core (1), and the concentric growth of layers with Fe depletion and Al (2) or Si (3) enrichment, which may account for carbonates (smithsonite or hydrozincite) and arsenates (aluminian scorodite or adamite).

In this French sample a zinc carbonate, probably smithsonite or hydrozincite, Zn_5_(CO_3_)_2_(OH)_6_, was tentatively identified by SEM-EDX analysis (Fig. 3-A4). This mineral phase can be the result of an oxidation step during (or following) azurite deposition. A secondary sulphide enrichment event is also hypothesised because of the presence of chalcopyrite (Fig. 3-A1), in nanometric spherule, and pyrite (Fig. 3-A2), in micrometric grains. A metal-poor aluminosilicate-rich outer zone gives evidence of Cu solubilization and the possible deposition of chrysocolla and quartz (Fig. 3-A3). Indeed, high internal variability can be attributed to a significant amount of impurities within the crystals from the same mine, the latter being the result of paragenesis, as pointed out by Aru and co-authors [11]. Zn is often present as inclusion in azurite in the form of smithsonite [17], a mineral that is known to occur in Chessy. The azurite quarry is called ‘Blue Mine’ and was formed by the circulation of acid water that dissolved the carbonate bed. Copper and zinc replaced calcium and/or magnesium by precipitating as carbonates and leading to the formation of azurite, malachite and smithsonite [26]. Further evidence of this process is given by the binary graph showing the inverse proportionality between CuO and ZnO in the French samples (Fig. 2B). The lowest value of CuO, 67%, corresponds to the maximum concentration of ZnO, 4.408%, demonstrating how a substitution of Cu could have occurred causing the precipitation of Zn as carbonate during the oxidation step.

In the case of Chessy, variability is seen both between and within samples. Particularly, for the specific sample 13238/51 SEM-EDX showed the unusual presence of Sn and In, along with Cu. Indeed, tin and indium have been recently documented in the nearby deposit of Charrier, described as the result of the interaction between magmatic Sn–Bi–In fluids and a volcano-sedimentary sequence [27].

SEM-EDX also revealed that a Fe–Si–Al association is commonplace, as it was seen in samples from Cornwall, Laurion and Siegen.

### 3.2 Trace elements

**Fig. 4:**
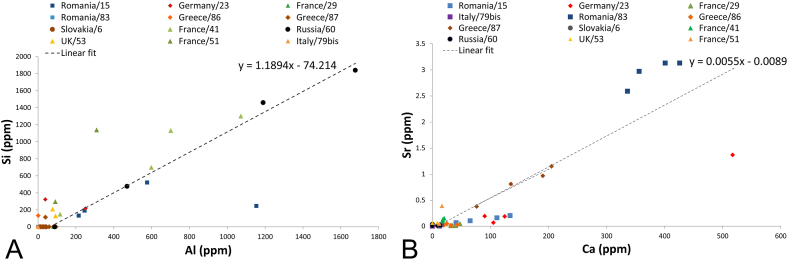
Correlation between the contents of A) Si and Al, B) Ca and Sr in each of the samples analysed; the last two digits in the legend correspond to the last digits of the Museum code in Table 1; linear fit for direct proportionality is shown in grey.

LA-ICP-MS analysis (Table S2) confirmed that the sample from Russia has the highest content of Al, but also of Si. The positive correlation between Al and Si concentration (Fig. 4A) was also verified for other samples: from France (13228/41) and Romania (13202/15). This trend can be explained by the presence of clay minerals, while an inverse correlation can be due to quartz (as found for the Cornwall sample). Indeed, both mineral phases are said to occur in association with azurite [18]. Some samples – from Germany, Greece and Slovakia, but also the French 13216/29, showed detectable Al but not Si.

Conversely, in the sample from Slovakia Al seems to correlate with Fe, both within more homogeneous areas and those containing impurities. SEM-EDX showed that the latter features concentric growth and high iron content in the core (Fig. 3B). Fe depletion progressively occurs in the outer circular layers with Al enrichment. As, with minor Zn and Cu are also present. Such features may account for the association between arsenates, like aluminian scorodite, (Fe^3+^,Al)AsO_4_· 2H_2_O, or adamite, Zn_2_(AsO_4_) (OH), and carbonates such as smithsonite or hydrozincite [28]. Both are common minerals to occur with azurite and malachite in oxidation zones of copper ore deposits [16].

A positive correlation was also verified between Ca and Sr (Fig. 4B), which is particularly meaningful in one sample from Romania, 13202/15, and one from Greece, 16696/87. Although the correlation between Ca and Sr may be due to sulphates, in azurite it is more likely to come from calcium-carbonate species, and their tendency to include high Sr content from seawater during their formation [29]. Indeed, calcite has been documented in samples from the Kingdom of Hungary [11]. In our study, the samples from Romania show the highest concentration of Sr (0.06–3.13 ppm), followed by Germany (max 1.37 ppm) and Greece (max 1.15 ppm). Lavrion rocks are in fact known to foster groundwater movement due to both lithological features and local tectonic structures [28]. Fig. 4B also shows the notable variability in both Ca and Sr contents among the samples from the same ore.

In agreement with EMPA data, Zn has quite high concentration in different localities: one sample from France (13228/41, 0.36–2.19%), Romania (0.34–0.40%) and Slovakia (0.14–0.28%). As already suggested, the element might come from Zn arsenates or carbonates. Although the concurrent presence of Zn and As was observed by SEM-EDX in some impurities of the French samples, we could not verify the presence of Bi, and hence confirm whether As is rather present in the form of mixite, a mineral that has already been identified with azurite in a 14th-century painting [14]. In the present study, the highest concentration of As was found in one sample from Greece (sample 16696/87, 0.15–0.39%) and one from Romania (sample 14890/83, 0.13–0.19%). Minor amount of As was also detected in the sample from Slovakia (48–195 ppm).

Lead shows high variability within the same locality. For example, in one Romanian sample the concentration ranges from 595 to 1497 ppm (13202/15), while in the other (14890/83) it is lower than 105 ppm. The detection of Pb can be linked to the presence of cerussite, although this mineral phase has never been documented in ‘Banat’. Nor it has for Chessy, which has the highest Pb content in our study (except for sample 13238/51), showing 839–2330 ppm for sample 13216/29 and 88–597 ppm for 13228/41. However, lead concentration is too low to be verified and attributed to any specific mineral phase by SEM-EDX. Sample 13238/51 is also the only one with moderate Sn content (40–244 ppm), in agreement with SEM-EDX results.

As previously documented [11,16,17], Fe is frequently detected in association with copper carbonates, in the form of iron oxides or hydroxides, but also sulphates. The highest Fe content was found in the French samples, with great variability among the three samples or within the same crystal (7–630 ppm). The presence of Fe is also consistent with the description provided by MUST, where the presence of yellow ochre is described in all the French samples. Moderate content was also found in the sample from Germany (12–168 ppm). Although Fe concentration was quite low in LA-ICP-MS results for the Greek sample 16696/87, SEM-EDX showed that some porous aggregates form a matrix between crystals of azurite and they contain Fe and Si.

Titanium is mostly representative of two out-of-three samples from France, 13228/41 and 13238/51 (0.04–4 ppm). Low content of Li (0.1–0.2 ppm) and V (0.04–3.11 ppm) was found in these French samples only, while the highest Mn content was recorded in the third French sample (13216/29, 28–96 ppm).

Although EMPA showed that some Ba is present (0.03–0.28%), in LA-ICP-MS analyses Ba was most often below 0.1 ppm. It is possible that the hand-picking carried out to prepare the cross-sections excluded any barite crystals, which are frequently described in association with azurite, even on paintings [15] and illuminated manuscripts [18], and that the laser did not focus on any area with barium. The only exception was the sample from Chessy 13238/51 (0.5–4.6 ppm).

**Fig. 5:**
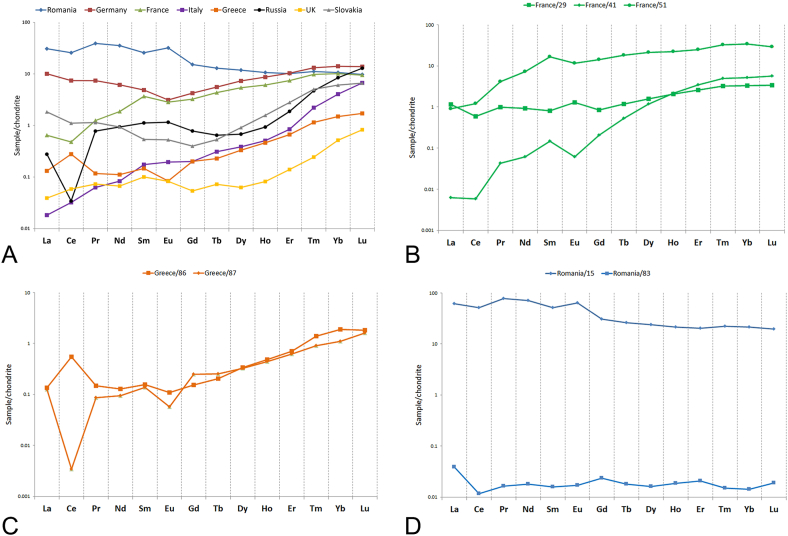
Representative chondrite-normalised [25] REE fractionation patterns from LA-ICP-MS data: A) average concentrations for each locality; REE plot of each analysed point in the samples from B) France, C) Greece, D) Romania.

REE offered further criteria for the discrimination of the azurite samples. Most of the chondrite-normalised [30] REE patterns (Fig. 5) show low-to-moderate enrichment in heavy rare earth elements (HREE), which may reflect the composition of the water coming in contact with azurite during and after its formation. The only exception is represented by the samples from Romania, which in turn have slight enrichment in light REE (LREE), the highest abundance in REE, negative Ce and positive Eu anomaly (Fig. 5D). The strongest Ce anomaly is shown by the average pattern of the sample from Russia, possibly suggesting the interaction with seawater (Fig. 5A). A strong Ce anomaly was also found for the Greek sample 16696/87 (Fig. 5C). However, when this pattern is averaged with the one from the second Greek sample, the anomaly turns slightly positive.

**Fig. 6:**
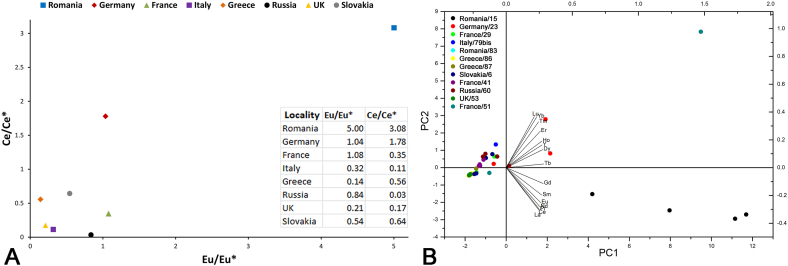
A) Average anomalous Eu and Ce behaviour for each locality, quantified using the parameters Eu/Eu* (x) and Ce/Ce* (y); B) PC diagram (first 2 PCs, 98% of the total variance) calculated from REE content.

A negative Eu anomaly was recorded for Greece, Germany and France (Fig. 5B). The highest anomalies of both elements are recorded for Romania and Germany. The binary plot comparing Eu/Eu* and Ce/Ce* (Fig. 6A) offers a criterion for the discrimination of these localities.

Variations in the REE patterns occur within each locality. Among the French samples, 13216/29 is the only one with moderately positive Eu anomaly. REE patterns of 13228/41 (with higher fractionation, HREE enrichment), and 13238/51 might tell about the interaction with hydrothermal water. The difference in absolute abundance between these two samples can be related to pH variations, where sample 13238/51 might have interacted with more acidic water [31].

When PCA was performed on REE data (Fig. 6B), it showed that the first four PCs account for 99.8% of the total variance. The score plot with loadings suggests that HREE can be used to discriminate the sample from Germany and one from France (13238/51), while LREE help the separation of one sample from Hungary (13202/15).

## 4 Provenance criteria

Aluminium, silicon and iron seem to have potential in the discrimination of some localities, especially the French one. However, these elements are quite common on paintings, as they might come from other pigments used in mixture with azurite or in adjacent paint layers; hence, they cannot be considered markers for provenance.

Nevertheless, it may be significant to consider the results regarding the sample 13247/60 from Russia which is characterized by particularly high Al and Si contents; these elements can stand for the presence of clay minerals that distinguish the geology of the Siberian mine [32,33]. Further investigations even on additional samples from Solotuschinsk could confirm this hypothesis.

Calcite is also a frequent occurrence in paintings where azurite is used. However, Sr might be a diagnostic element to prove that carbonate species are linked to a specific provenance of the blue pigment, from Romania and/or Greece. One Greek sample showed high Sr content and strong negative Ce anomaly. Both features suggest the involvement of seawater in the genesis of the azurite from Laurion. The level of concentration of Sr in our samples is not high enough to be detected by any portable instrumentation, but it can be reasonably identified by a benchtop instrument used for the analysis of trace elements in micro-samples.

Zinc is a marker for France and the Kingdom of Hungary (Romania and Slovakia). In the samples from these localities Zn concentration is well above the sensitivity of portable XRF systems, explaining why this element has been frequently documented during the technical analysis of artworks. This is the first time that Zn is directly associated with azurite from specific localities, although its presence had been previously detected in azurite-rich areas of illuminated manuscripts [17] or wall paintings [16]. More recently, Zn has been linked to azurite in the ‘Adoration of the Kings’ by Botticelli and Filippino Lippi, in the collection of The National Gallery, London. It has been pointed out using macro-XRF with reflectance imaging spectroscopy (RIS) in the short-wave IR region (SWIR) that in the *Adoration* azurite may come from different batches. RIS-SWIR and XRF maps of Cu, Ba and Zn, and the analysis of a cross-section by SEM-EDX, proved that there are two types of azurite, one which contains barium sulphate and one with zinc only [34]. The pigments would probably have been selected just for their colour, and the artists were likely unaware that the pigments they were using had slightly different composition and probably came from different geological sources.

As Zn does not correlate with As in our samples, it might be speculated that they are due to different mineral impurities, such as smithsonite and mixite. However, as we could not detect the presence of Bi by SEM-EDX, a zinc carbonate seems the most likely accessory phase in some areas of the ‘Kingdom of Hungary’. In the Slovakian sample, Fe-containing arsenates occur along with Zn carbonates, as it has been documented in gossan or partly weathered sulphide ores [28].

On the other hand, Zn phases and their association with sulphides (chalcopyrite and pyrite) and metal-poor silicates (chrysocolla or quartz) seem to account for an oxidation and secondary sulphide enrichment process [25,35] in one of the French samples. However, further investigation might include the application of XRD or Raman analysis to confirm the presence of these mineralogical species.

It is worth mentioning that among the deposits investigated, Romania is the sole locality showing high content of both As and Zn (>0.2%). Zn and As impurities have been documented in cross-sections from blue areas of the 14th-century wall paintings in the Capitular Hall of the Benedictine monastery at Sázava, established in central Bohemia (Czech Republic) in 1032 [16]. The results of the present study might prove that the azurite used in the decorations has a local origin.

High variability within the same sample or locality is not surprising and it is due to the paragenesis process. Indeed, Smieska and co-authors pointed out that dramatic chemical variations in azurite can occur within the same manuscript illumination [18]. Overall, the samples from France seem to be the most representative of the internal variability within the same deposit. In particular, the two samples 13228/41 and 13238/51 show the strongest similarities in trace elements. Specifically, a very distinct REE pattern likely suggests that these samples had formed under the same environmental conditions and because of the action of hydrothermal water. These were among the few samples to show moderate Ti content. To the authors’ knowledge, previous references do not mention any evidence of Ti-bearing minerals associated with azurite from Chessy, possibly because its content is usually below the detection limit of the technique used in those studies. This element probably comes from minerals like anatase or rutile that are commonly associated with azurite in other localities, such as Hungary, UK and Siberia [11]. Our investigation has demonstrated the presence of Ti in the samples from Chessy and how French supplies cannot be excluded when this element is detected with azurite in paintings. For the French samples, it has been also highlighted that heterogeneity is high within the deposit. Specifically, a quite rare association between Sn, In and Cu might give evidence of the westernmost area, which possibly shares similarities with the nearby Charrier (Allier) ore deposit [27].

Previous studies have pointed out the association of both Ba and S with azurite. However, these two elements and their concentrations are not related in any case in our samples, and Ba content is often below the limit of detection. Therefore, it was not possible to verify the presence of barite, which has been frequently reported as an impurity in paint layers with azurite [15,16,34]. We can hypothesize that the absence of barite is the result of the purification process carried out before embedding our samples. This might imply that barite does not occur within a single azurite crystal, but more likely as a separate phase, residual after pigment purification in the artist's workshop. Sulphur may be linked to the presence of copper sulphides and sulfosalts from which azurite is formed.

A tentative match between our results and the minerals and elements identified in previous studies is hereby provided, along with the type of artwork and the analytical techniques used to identify azurite and its associated impurities (Table 2).

**Table 2:** Chemical markers identified in the present study for the provenance of azurite and tentative match with previous studies, along with type of artwork and analytical techniques used to detect associated minerals/chemical elements (additional details in Figs. S4–S11; in bold are the elements that might also come from other geological environments or pigments used in mixture with azurite on paintings). Countries abbreviations are: FR = France, GE = Germany, GR = Greece, SL = Slovakia, IT = Italy, RO = Romania, RU = Russia, UK = United Kingdom.

Locality	Possible chemical markers	Fig.	Evidence in mineral samples	Evidence in artworks	Techniques
Cornwall UK	(<0.1%) **Fe**, Zn, **Si**, **Al** (<100 ppm) **K**, **Pb**, **Ca**, **Ti**, Zr, **Mn**, As	S4	Goethite, hematite, quartz, cerussite [11,14]	• *Madonna and Child*, panel painting (Giotto, original location unknown, c. 1310/1315), Samuel H. Kress Collection, National Gallery of Art, Washington, D.C [14]	μ-Raman sp. SEM-EDX EBSD
				• *Christ in Majesty with Twelve Apostles*, illuminated manuscript leaf (workshop of Pacino di Buonaguida, Florence, c. 1320) [17]	Multisp. imaging XRF FORS
Campiglia IT	(<0.1%) Zn (<80 ppm) **Pb**, **Ca**, As, **Mn**	S5	–	–	–
Siegen GE	(<900 ppm) **Ca**, **Si**, **Al**, **Fe**, Y (<100 ppm) Zn, Pb, Ba, Sr, Ni, Mn, Co	S6	Goethite, hematite, cerussite, beudantite [11]	• Italian, Spanish, French, Dutch illuminated manuscript leaves (13th-15th cent.), Cornell University Library Rare and Manuscript Collection [19]	p-XRF, SR-XRF SR-XRD
Chessy FR	(<4%) Zn, **Pb**, **Al**, **Si**, **Fe** (<300 ppm) Sn, **Ca**, As, **K**, **Ti**, V, **Na**, U, Be	S7	Goethite, hematite, quartz, calcite [11], smithsonite [26]	• *Christ in Majesty with Twelve Apostles*, illuminated manuscript leaf (workshop of Pacino di Buonaguida, Florence, c. 1320) [17]	Multisp. imaging XRF FORS
				• *Adoration of the Kings,* panel painting *(*S. Botticelli and F. Lippi, Florence, c. 1470), The National Gallery, London [34]	Macro-XRF
Wolwodina RO	(<2%) Zn, As, **Pb**, **Al**, **Ca**, **Si** (<150 ppm) Sr, Fe, Mn, K, Ni, Be	S8	Goethite, hematite, calcite, quartz, rhodochrosite [11]	• *The Betrothal of Mary and Joseph*, *The Joseph's Doubt*, *The Nativity*, wall paintings, Capitular Hall, Sázava Monastery, Sázava, Czech Republic [16]	Raman sp. SEM-EDX XRD
				• *The ‘Griselda panels’,* series of 3 panel paintings (anonymous master), The National Gallery, London [34] a	
Gollnitz SK	(<1%) Zn (<500 ppm) As, **Al**, **Ca**, **Fe**	S9	Mixite	• *Madonna and Child*, panel painting (Giotto, original location unknown, c. 1310/1315), Samuel H. Kress Collection, National Gallery of Art, Washington, D.C [14]	μ-Raman sp. SEM-EDX EBSD
Laurion GR	(<1%) As (<100 ppm) **Ca**, Zn, **Si**, **Al** (<40 ppm) **Mn**, **Fe**, Ni, **Pb**, Co, Sr, Be	S10	–	–	
Solotuschinsk RU	(<0.4%) **Si**, **Al**, Zn (<140 ppm) **Pb**, **Fe**, **Ca**, As, **K**	S11	Goethite, hematite, cerussite, jarosite [11]	–	μ-Raman sp.

a It shall be remarked that the authors mention Schwaz (Tyrol) as the most plausible source [36], because ‘a distinctive yellowish-green complex copper mineral’ with Cu, Zn, Sb and As was detected by EDX analysis in association with dolomite (see note 41 therein), and both occur in historic copper and silver mines in this region. We could not analyse any mineral sample from this region, nor measure Sb content; however, we suggest Romania as an alternative possible source based on Zn and As co-occurrence.

## 5 Conclusions

This study provides insight on the chemical variability, at major, minor and trace element level, of azurite from historically meaningful localities. It has been proved that this blue pigment can show compositional variations even within the same artwork, suggesting that artists may have used different azurite supplies at the same time. Although the small number of samples prevents from drawing any broad conclusion, the present research provides a contribution to the understanding of workshop practises and pigment trades. Notwithstanding the limitations given by the sample size, the results obtained in this study are promising and clarifies the characteristics and differences of the investigated historical supplies. A direct association of Zn-containing azurite with the French supply was highlighted. Similarly, As was linked to samples from France and Romania. This element is probably present in the form of a zinc carbonate. We could not verify the presence of mixite, a mineral that has already been identified in association with azurite from ‘Banat’. The high content of Si and Al detected in the Siberian sample suggests the presence of clay minerals; the latter are often found in pigment mixtures when azurite is directly analysed on paintings, making it difficult to use Si and Al as provenance markers. The correlation between Ca and Sr seems to demonstrate that the carbonate species are linked to a specific origin of the mineral, *i.e.* Romania and Greece.

REE can provide further characterization. In detail, Ce and Eu seem to be meaningful for the discrimination of localities from Germany and Romania. PCA processing of REE data is proposed to highlight relationships and differences among the investigated samples based on enrichment of HREE or LREE.

Further investigation might link the chemical impurities found in this study with the corresponding minerals using Raman spectroscopy or X-ray diffraction. The impact of any purification process used to make the mineral suitable for painting should be elucidated in the future. A systematic scrutiny of azurite associations in works of art, either with non-destructive techniques or micro-sampling, could be usefully explored in further research. Bringing to fruition a more comprehensive chemical and mineralogical dataset may eventually explain the use of different supplies in the same painting. This could be due to commercial reasons, for example the end of provisions/trade agreements, or to technical aspects, like better quality, purity or hue of the pigment from a certain locality.

## Author contribution statement

S. Capriotti: Performed the experiments; Analyzed and interpreted the data; Wrote the paper.

L. Medeghini; S. Mignardi: Contributed reagents, materials, analysis tools or data; Wrote the paper.

M. Petrelli: Conceived and designed the experiments; Performed the experiments; Contributed reagents, materials, analysis tools and data; Wrote the paper.

M. Botticelli: Conceived and designed the experiments; Analyzed and interpreted data; Wrote the paper.

## Data availability statement

Data included in article/supp. material/referenced in article.

## Declaration of competing interest

The authors declare the following financial interests/personal relationships which may be considered as potential competing interests: Michela Botticelli reports financial support was provided by University of Rome La Sapienza.
