# Supplementary material for: The blue road: Provenance study of azurite samples from historical locations through the analysis of minor and trace elements
Source: Heliyon. 2023 Aug 11;9(8):e19099. doi: 10.1016/j.heliyon.2023.e19099 (PMC10469567; doi:10.1016/j.heliyon.2023.e19099)
Supplement: Multimedia component 1 [file mmc1.docx]

# **Supplementary**

**Table S1**. Concentration of major oxide (wt%) given by EMPA (number of analyses n>3), with average value and standard deviation for each azurite sample; countries abbreviations as in Table 2

| **Sample** | **Fragment** | **Analysis point** | **SO_2_** | **Al_2_O_3_** | **MgO** | **CaO** | **CuO** | **ZnO** | **BaO** |
| --- | --- | --- | --- | --- | --- | --- | --- | --- | --- |
| **13240/53 Cornwall,**  **UK** |  | Right rim | n.d. | 0.01 | n.d. | 0.02 | 72 | 0.01 | 0.2 |
|  |  | Left rim | n.d. | 0.06 | n.d. | 0.02 | 72 | n.d. | 0.2 |
|  |  | Core | 0.002 | 0.01 | 0.01 | 0.01 | 71 | n.d. | 0.0 |
|  |  | Top rim | 0.01 | 0.03 | 0.001 | 0.03 | 71 | 0.04 | 0.1 |
|  | **I** | Bottom rim | n.d. | 0.004 | 0.02 | 0.004 | 72 | 0.03 | 0.2 |
|  | **Average** | | 0.003 | 0.02 | 0.01 | 0.02 | 72 | 0.02 | 0.2 |
|  | **Standard deviation** | | 0.01 | 0.02 | 0.01 | 0.01 | 0.4 | 0.02 | 0.04 |
| **13466/79bis Campiglia,**  **IT** |  | Top rim | 0.03 | 0.03 | 0.02 | 0.02 | 73 | 0.21 | 0.2 |
|  |  | Core | 0.02 | 0.004 | 0.02 | 0.03 | 73 | 0.13 | 0.2 |
|  | **I** | Bottom rim | 0.04 | 0.01 | n.d. | n.d. | 73 | n.d. | 0.2 |
|  |  | Top rim | 0.02 | 0.02 | n.d. | 0.03 | 73 | 0.18 | 0.2 |
|  |  | Core | 0.01 | 0.02 | n.d. | n.d. | 73 | n.d. | 0.1 |
|  | **II** | Bottom rim | 0.02 | 0.03 | n.d. | 0.01 | 73 | n.d. | 0.2 |
|  | **Average** | | 0.02 | 0.02 | 0.01 | 0.01 | 73 | 0.09 | 0.2 |
|  | **Standard deviation** | | 0.01 | 0.01 | 0.01 | 0.01 | 0.3 | 0.10 | 0.03 |
| **132107/23 Siegen,**  **GE** |  | Top rim | n.d. | 0.02 | n.d. | 0.03 | 73 | 0.02 | 0.1 |
|  |  | Core | 0.04 | 0.02 | 0.004 | 0.01 | 71 | 0.06 | 0.2 |
|  | **I** | Bottom rim | 0.01 | 0.03 | 0.01 | 0.02 | 72 | n.d. | 0.2 |
|  |  | Top rim | 0.02 | 0.004 | 0.04 | n.d. | 72 | 0.02 | 0.1 |
|  |  | Core | 0.03 | 0.02 | 0.02 | 0.03 | 72 | 0.06 | 0.1 |
|  | **II** | Bottom rim | n.d. | 0.05 | 0.01 | 0.04 | 71 | n.d. | 0.1 |
|  | **Average** | | 0.02 | 0.02 | 0.01 | 0.02 | 72 | 0.03 | 0.1 |
|  | **Standard deviation** | | 0.02 | 0.02 | 0.01 | 0.02 | 0.6 | 0.03 | 0.03 |
| **13216/29**  **Chessy,**  **FR** |  | Top rim | 0.04 | n.d. | 0.004 | 0.01 | 70 | 1.0 | 0.1 |
|  |  | Core | 0.2 | 0.03 | n.d. | 0.01 | 71 | 0.4 | 0.2 |
|  | **I** | Bottom rim | 0.10 | 0.03 | n.d. | 0.01 | 71 | 0.6 | 0.2 |
|  |  | Top rim | 0.04 | 0.02 | n.d. | 0.001 | 67 | 4 | 0.1 |
|  |  | Core | 0.01 | 0.06 | 0.04 | 0.03 | 69 | 1 | 0.2 |
|  | **II** | Bottom rim | 0.04 | 0.01 | 0.02 | n.d. | 71 | 0.7 | 0.1 |
|  |  | Top rim | 0.02 | n.d. | 0.01 | 0.02 | 70 | 0.9 | 0.1 |
|  | **III** | Bottom Rim | 0.01 | 0.04 | n.d. | 0.02 | 70 | 1 | 0.1 |
|  | **Average** | | 0.06 | 0.02 | 0.01 | 0.01 | 70 | 1 | 0.1 |
|  | **Standard deviation** | | 0.07 | 0.02 | 0.01 | 0.01 | 1 | 1 | 0.03 |
| **13228/41 Chessy,**  **FR** |  | Top left rim | n.d. | 0.2 | 0.03 | 0.01 | 72 | n.d. | 0.2 |
|  |  | Core | n.d. | 0.02 | 0.04 | 0.02 | 73 | n.d. | 0.2 |
|  | **I** | Bottom rim | 0.00 | 0.07 | 0.03 | n.d. | 72 | n.d. | 0.2 |
|  |  | Top left rim | n.d. | 0.03 | n.d. | 0.03 | 71 | n.d. | 0.1 |
|  |  | Core | 0.03 | 0.03 | 0.004 | 0.01 | 72 | n.d. | 0.1 |
|  | **II** | Bott. Right rim | n.d. | 0.01 | 0.01 | 0.01 | 72 | n.d. | 0.2 |
|  | **Average** | | 0.01 | 0.05 | 0.02 | 0.01 | 72 | n.d. | 0.2 |
|  | **Standard deviation** | | 0.01 | 0.06 | 0.02 | 0.01 | 1 | n.d. | 0.05 |
| **13238/51 Chessy,**  **FR** |  | Top rim | 0.05 | 0.03 | 0.01 | 0.02 | 71 | n.d. | 0.1 |
|  |  | Bottom rim | n.d. | n.d. | 0.01 | 0.003 | 72 | n.d. | 0.1 |
|  |  | Core | 3 | 0.02 | n.d. | 0.01 | 72 | n.d. | 0.2 |
|  |  | Left rim | 0.4 | 0.03 | 0.02 | 0.01 | 73 | n.d. | 0.2 |
|  | **I** | Right rim | 0.02 | n.d. | n.d. | 0.03 | 72 | 0.03 | 0.1 |
|  | **Average** | | 0.7 | 0.02 | 0.01 | 0.01 | 72 | 0.01 | 0.1 |
|  | **Standard deviation** | | 1 | 0.02 | 0.01 | 0.01 | 0.5 | 0.01 | 0.1 |
| **14890/83**  **Wolwodina,**  **RO** |  | Top rim | 0.05 | n.d. | 0.01 | 0.02 | 73 | 0.03 | 0.2 |
|  |  | Core | n.d. | 0.05 | 0.03 | 0.07 | 72 | 0.04 | 0.2 |
|  | **I** | Bottom rim | n.d. | 0.03 | 0.04 | 0.03 | 72 | 0.04 | 0.2 |
|  |  | Top rim | n.d. | 0.02 | 0.02 | 0.04 | 73 | n.d. | 0.2 |
|  |  | Core | n.d. | 0.03 | n.d. | 0.05 | 72 | 0.01 | 0.2 |
|  | **II** | Bottom rim | n.d. | 0.02 | n.d. | 0.06 | 73 | 0.01 | 0.1 |
|  |  | Top rim | n.d. | 0.08 | 0.003 | 0.05 | 72 | n.d. | 0.2 |
|  |  | Core | n.d. | n.d. | 0.01 | 0.03 | 71 | 0.1 | 0.1 |
|  | **III** | Bottom rim | n.d. | 0.02 | 0.02 | 0.07 | 71 | 0.2 | 0.1 |
|  | **Average** | | 0.01 | 0.03 | 0.01 | 0.05 | 72 | 0.05 | 0.2 |
|  | **Standard deviation** | | 0.02 | 0.03 | 0.01 | 0.02 | 0.6 | 0.07 | 0.03 |
| **13202/15**  **Wolwodina,**  **RO** |  | Top left rim | 0.1 | 0.1 | 0.02 | 0.02 | 71 | 0.3 | 0.2 |
|  |  | Right rim | 0.10 | 0.3 | n.d. | 0.03 | 71 | 0.5 | 0.1 |
|  | **I** | Core | 0.05 | 0.03 | 0.02 | 0.03 | 71 | 0.5 | 0.2 |
|  | **II** | Top rim | 0.08 | 0.03 | n.d. | 0.01 | 73 | 0.09 | 0.2 |
|  |  | Bottom rim | 0.09 | 0.1 | n.d. | 0.02 | 72 | 0.2 | 0.2 |
|  |  | Core | 0.1 | 0.02 | n.d. | 0.01 | 72 | 0.2 | 0.2 |
|  | **Average** | | 0.09 | 0.10 | 0.01 | 0.02 | 72 | 0.3 | 0.2 |
|  | **Standard deviation** | | 0.02 | 0.10 | 0.01 | 0.01 | 0.8 | 0.2 | 0.03 |
| **13193/6 Gollnitz,**  **SK** |  | Bottom rim | n.d. | 0.02 | n.d. | n.d. | 71 | 0.2 | 0.2 |
|  |  | Bottom rim | 0.01 | 0.05 | 0.01 | 0.03 | 72 | 0.2 | 0.2 |
|  |  | Core | 0.04 | 0.04 | n.d. | n.d. | 72 | n.d. | 0.2 |
|  | **I** | Left rim | n.d. | 0.07 | 0.02 | 0.01 | 72 | 0.2 | 0.2 |
|  |  | Right Rim | 0.03 | 0.02 | n.d. | 0.02 | 71 | 0.1 | 0.2 |
|  |  | Left rim | n.d. | 0.004 | 0.001 | 0.02 | 72 | 0.1 | 0.1 |
|  | **II** | Core | n.d. | 0.04 | 0.001 | 0.01 | 71 | 0.1 | 0.1 |
|  |  | Top rim | n.d. | 0.03 | 0.02 | 0.02 | 72 | 0.9 | 0.1 |
|  |  | Core | 0.01 | 0.03 | n.d. | n.d. | 73 | 0.2 | 0.1 |
|  | **III** | Bottom rim | n.d. | 0.03 | n.d. | 0.01 | 72 | 0.4 | 0.1 |
|  | **Average** | | 0.01 | 0.03 | 0.01 | 0.01 | 72 | 0.2 | 0.1 |
|  | **Standard deviation** | | 0.02 | 0.02 | 0.01 | 0.01 | 0.7 | 0.2 | 0.1 |
| **16696/87**  **Laurion,**  **GR** |  | Bott. left rim | 0.02 | 0.03 | n.d. | 0.03 | 72 | n.d. | 0.1 |
|  |  | Top right rim | n.d. | n.d. | 0.03 | 0.03 | 71 | n.d. | 0.3 |
|  | **I** | Core | 0.02 | 0.01 | n.d. | 0.03 | 72 | 0.01 | 0.2 |
|  |  | Top rim | n.d. | 0.04 | n.d. | 0.01 | 73 | 0.01 | 0.2 |
|  |  | Core | 0.02 | 0.02 | n.d. | 0.02 | 73 | 0.07 | 0.1 |
|  | **II** | Bottom rim | 0.00 | 0.10 | 0.02 | 0.03 | 72 | n.d. | 0.2 |
|  | **Average** | | 0.01 | 0.03 | 0.01 | 0.03 | 72 | 0.01 | 0.2 |
|  | **Standard deviation** | | 0.01 | 0.03 | 0.01 | 0.01 | 0.7 | 0.03 | 0.1 |
| **16695/86**  **Laurion,**  **GR** |  | Top rim | n.d. | 0.04 | n.d. | 0.02 | 72 | n.d. | 0.1 |
|  |  | Bottom rim | 0.02 | 0.02 | n.d. | n.d. | 73 | n.d. | 0.2 |
|  | **I** | Core | n.d. | n.d. | 0.01 | 0.01 | 72 | n.d. | 0.1 |
|  | **Average** | | 0.01 | 0.02 | 0.004 | 0.01 | 72 | n.d. | 0.1 |
|  | **Standard deviation** | | 0.01 | 0.02 | 0.01 | 0.01 | 0.2 | n.d. | 0.04 |
| **13247/60**  **Solotuschinsk, RU** |  | Top rim | 0.01 | 1.0 | 0.05 | 0.02 | 71 | 0.08 | 0.1 |
|  |  | Bottom rim | 0.03 | 0.05 | n.d. | 0.01 | 72 | 0.09 | 0.2 |
|  | **I** | Core | 0.02 | 0.2 | 0.01 | n.d. | 72 | 0.09 | 0.1 |
|  |  | Top rim | n.d. | 0.7 | n.d. | 0.001 | 70 | 0.09 | 0.1 |
|  |  | Core | n.d. | 0.07 | 0.03 | 0.01 | 73 | 0.01 | 0.1 |
|  | **II** | Bottom rim | 0.01 | 0.03 | 0.01 | n.d. | 72 | n.d. | 0.1 |
|  | **Average** | | 0.01 | 0.3 | 0.02 | 0.01 | 71 | 0.06 | 0.1 |
|  | **Standard deviation** | | 0.01 | 0.40 | 0.02 | 0.01 | 0.8 | 0.04 | 0.05 |

**Table S2**. LA-ICP-MS data (ppm) for major, minor and trace elements in all the analysed points of the azurite samples (Countries abbreviations as in Table 2), with LOD calculated according to [24]

| **Sample** |  | **Li** | **Be** | **Na** | **Al** | **Si** | **K** | **Ca** | **Ti** | **V** | **Mn** | **Fe** | **Co** | **Ni** | **Zn** | **Ga** | **As** | **Rb** | **Sr** | **Zr** | **Nb** | **Sn** | **Ba** | **Hf** | **Pb** | **Th** | **U** |
| --- | --- | --- | --- | --- | --- | --- | --- | --- | --- | --- | --- | --- | --- | --- | --- | --- | --- | --- | --- | --- | --- | --- | --- | --- | --- | --- | --- |
| **LOD [24]** |  | 0.05 | 0.01 | 3 | 0.2 | 122 | 0.5 | 9 | 0.2 | 0.02 | 0.1 | 1 | 0.02 | 0.05 | 0.09 | 0.01 | 0.05 | 0.01 | 0.001 | 0.002 | 0.002 | 0.01 | 0.004 | 0.001 | 0.005 | 0.0002 | 0.0003 |
| **13202/15 Wolwodina, RO** |  |  | 0.04 |  | 215 | 132 |  | 41 |  |  | 1 |  |  | 0.2 | 3510 | 0.3 | 4 |  | 0.06 | 0.005 | 0.003 | 0.02 |  | 0.002 | 595 | 0.005 | 2 |
|  |  |  | 0.05 |  | 246 | 191 | 0.9 | 65 |  |  | 1 |  |  | 0.2 | 4040 | 0.5 | 3 |  | 0.1 | 0.006 | 0.003 | 0.02 | 0.005 | 0.007 | 653 | 0.002 | 2 |
|  |  |  | 0.05 |  | 576 | 522 | 0.8 | 111 |  |  | 1 |  |  | 0.6 | 3360 | 0.6 | 5 |  | 0.2 | 0.009 | 0.005 | 0.02 | 0.008 | 0.011 | 813 | 0.02 | 2 |
|  |  |  | 0.06 |  | 1154 | 246 | 1.8 | 134 |  |  | 1 |  |  | 1.0 | 3876 | 0.6 | 15 | 0.02 | 0.2 | 0.007 | 0.003 | 0.02 | 0.02 | 0.008 | 1497 | 0.1 | 8 |
|  | **Mean** |  | 0.05 |  | 548 | 273 | 1 | 88 |  |  | 1 |  |  | 0.5 | 3697 | 0.5 | 7 | 0.02 | 0.1 | 0.007 | 0.003 | 0.02 | 0.01 | 0.007 | 890 | 0.04 | 3 |
|  | **Std dev** |  | 0.006 |  | 378 | 149 | 0.4 | 37 |  |  | 0.2 |  |  | 0.3 | 273 | 0.1 | 5 | 0.000 | 0.05 | 0.001 | 0.001 | 0.003 | 0.006 | 0.003 | 360 | 0.05 | 2 |
| **132107/23 Siegen,**  **GE** |  |  | 0.4 |  | 38 | 323 |  | 124 |  |  | 0.1 | 12 | 0.03 | 0.08 | 6 |  |  |  | 0.2 | 0.2 | 0.006 | 0.02 | 0.3 | 0.003 | 0.5 | 0.003 | 0.009 |
|  |  |  | 0.6 |  | 250 | 217 |  | 517 |  |  | 0.3 | 52 | 0.07 | 1 | 17 | 0.1 |  |  | 1 | 0.2 | 0.005 | 0.03 | 2 | 0.003 | 4 | 0.02 | 0.09 |
|  |  |  | 0.8 |  | 11 |  |  | 105 |  |  | 0.8 | 168 | 0.4 | 0.9 | 15 | 0.06 |  |  | 0.07 | 0.1 | 0.003 | 0.02 | 0.09 | 0.004 | 0.009 | 0.03 | 0.06 |
|  |  |  | 0.4 |  | 79 |  |  | 90 |  |  | 0.4 | 21 | 0.03 |  | 11 | 0.03 |  |  | 0.2 | 0.03 |  | 0.02 | 0.4 |  | 2 | 0.004 | 0.03 |
|  | **Mean** |  | 0.5 |  | 95 | 270 |  | 209 |  |  | 0.4 | 63 | 0.1 | 0.7 | 12 | 0.07 |  |  | 0.5 | 0.1 | 0.005 | 0.02 | 0.8 | 0.003 | 2 | 0.01 | 0.05 |
|  | **Std dev** |  | 0.2 |  | 93 | 53 |  | 178 |  |  | 0.2 | 62 | 0.2 | 0.4 | 4 | 0.03 |  |  | 0.5 | 0.06 | 0.001 | 0.003 | 0.9 | 0.000 | 2 | 0.01 | 0.03 |
| **13216/29 Chessy,**  **FR** |  |  | 1 |  | 23 |  |  | 48 |  |  | 96 | 19 | 0.1 | 0.1 | 21900 |  | 0.2 |  | 0.05 | 0.008 | 0.002 | 0.01 | 0.03 |  | 839 | 0.003 | 0.01 |
|  |  |  | 0.3 |  | 85 |  |  | 40 |  |  | 38 | 46 | 0.1 |  | 6580 |  | 0.3 |  | 0.01 | 0.009 | 0.003 |  | 0.005 |  | 2330 | 0.001 | 0.04 |
|  |  |  | 0.2 |  | 80 |  |  | 35 |  |  | 28 | 68 | 0.09 |  | 3620 |  | 0.7 |  | 0.01 | 0.004 | 0.003 | 0.03 | 0.005 |  | 1970 | 0.007 | 0.04 |
|  |  |  | 0.2 |  | 45 |  |  | 32 |  |  | 30 | 33 | 0.1 | 0.06 | 6540 |  | 0.4 |  | 0.01 | 0.01 | 0.003 | 0.02 | 0.003 |  | 1420 | 0.009 | 0.02 |
|  | **Mean** |  | 0.4 |  | 58 |  |  | 38 |  |  | 48 | 42 | 0.1 | 0.09 | 9660 |  | 0.4 |  | 0.02 | 0.007 | 0.003 | 0.02 | 0.01 |  | 1640 | 0.005 | 0.03 |
|  | **Std dev** |  | 0.4 |  | 26 |  |  | 6 |  |  | 28 | 18 | 0.02 | 0.04 | 7168 |  | 0.2 |  | 0.01 | 0.002 | 0.000 | 0.007 | 0.01 |  | 565 | 0.003 | 0.01 |
| **13466/79bis Campiglia,**  **IT** |  |  |  |  |  |  |  |  |  |  | 0.2 |  |  |  | 1056.00 |  | 0.122 |  |  | 0.03 | 0.002 | 0.02 | 0.003 |  | 17 | 0.001 | 0.001 |
|  |  |  |  |  |  |  |  | 12 |  |  |  |  |  |  | 42.90 |  | 0.208 |  | 0.004 | 0.02 | 0.002 | 0.02 |  |  | 53 | 0.009 | 0.006 |
|  |  |  |  |  |  |  |  | 13 |  |  |  |  |  |  | 143.00 |  |  |  | 0.001 | 0.2 | 0.003 | 0.02 |  | 0.002 | 3 | 0.001 | 0.000 |
|  |  |  |  |  |  |  |  |  |  |  |  |  |  |  | 7.80 |  | 3.390 |  | 0.002 | 0.003 |  | 0.02 |  |  | 6 | 0.001 |  |
|  | **Mean** |  |  |  |  |  |  | 12 |  |  | 0 |  |  |  | 312 |  | 1 |  | 0 | 0 | 0 | 0 | 0 | 0 | 20 | 0 | 0 |
|  | **Std dev** |  |  |  |  |  |  | 0 |  |  | 0 |  |  |  | 432 |  | 2 |  | 0 | 0 | 0 | 0 | 0 | 0 | 20 | 0 | 0 |
| **14890/83 Wolwodina, RO** |  |  | 0.9 |  | 1 |  |  | 336 |  |  |  | 2 | 0.2 | 0.6 | 640 |  | 1990 |  | 3 | 0.004 | 0.004 | 0.02 | 0.04 |  | 84 |  | 0.008 |
|  |  |  | 0.4 |  | 1 |  |  | 356 |  |  |  | 3 | 0.1 | 0.4 | 442 |  | 1413 |  | 3 | 0.005 | 0.003 | 0.02 | 0.04 |  | 60 |  | 0.006 |
|  |  |  | 0.3 |  | 0 |  | 0.7 | 401 |  |  |  | 2 | 0.1 | 0.2 | 569 |  | 1360 |  | 3 | 0.005 | 0.003 | 0.02 | 0.03 |  | 104 |  | 0.005 |
|  |  |  | 0.3 |  | 0 |  |  | 426 |  |  |  | 2 | 0.1 | 0.2 | 598 |  | 1300 |  | 3 | 0.002 | 0.003 | 0.01 | 0.03 |  | 94 |  | 0.004 |
|  | **Mean** |  | 0.5 |  | 0.9 |  | 0.7 | 380 |  |  |  | 3 | 0.1 | 0.4 | 562 |  | 1516 |  | 3 | 0.004 | 0.003 | 0.02 | 0.03 |  | 85 |  | 0.006 |
|  | **Std dev** |  | 0.2 |  | 0.5 |  | 0.0 | 36 |  |  |  | 0.1 | 0.02 | 0.1 | 74 |  | 277 |  | 0.2 | 0.001 | 0.000 | 0.001 | 0.004 |  | 16 |  | 0.001 |
| **16695/86 Laurion,**  **GR** |  |  |  |  | 0 | 133 |  | 19 |  |  |  |  | 0.6 | 8 | 0.2 |  |  |  | 0.02 | 0.004 | 0.004 | 0.01 | 0.007 |  | 0.03 |  | 0.000 |
|  |  |  |  |  | 5 |  |  | 25 |  |  |  | 2 |  | 7 | 2 |  |  |  | 0.05 | 0.3 | 0.003 | 0.02 | 0.06 | 0.002 | 0.05 | 0.02 | 0.002 |
|  |  |  |  |  | 1 |  |  | 32 |  |  | 29 | 4 | 11 | 1 | 17 |  |  |  | 0.01 |  |  |  | 0.009 |  | 0.03 | 0.003 |  |
|  |  |  | 0.04 |  | 2 |  |  | 43 |  |  | 1 |  | 0.3 |  | 1.0 |  |  |  | 0.03 | 0.02 | 0.003 | 0.01 | 0.02 |  | 0.1 | 0.02 | 0.005 |
|  | **Mean** |  | 0.04 |  | 2 | 133 |  | 30 |  |  | 15 | 3 | 4 | 6 | 5 |  |  |  | 0.03 | 0.1 | 0.003 | 0.01 | 0.02 | 0.002 | 0.06 | 0.02 | 0.002 |
|  | **Std dev** |  | 0.000 |  | 2 | 0 |  | 9 |  |  | 14 | 1 | 5 | 3 | 7 |  |  |  | 0.01 | 0.1 | 0.000 | 0.001 | 0.02 | 0.000 | 0.03 | 0.009 | 0.002 |
| **16696/87 Laurion,**  **GR** |  |  | 0.05 |  | 58 |  |  | 205 |  |  |  | 2 | 0.2 | 0.06 | 109 | 0.01 | 2042 |  | 1 | 0.01 | 0.004 | 0.02 | 0.03 |  | 3 |  | 0.6 |
|  |  |  | 0.1 |  | 12 |  |  | 190 |  |  | 0.4 | 4 | 0.08 | 0.1 | 112 |  | 1466 |  | 1.0 | 0.03 | 0.003 | 0.02 | 0.02 |  | 3 | 0.001 | 0.4 |
|  |  |  | 0.3 |  | 39 | 114 |  | 135 |  |  |  | 7 | 0.05 | 0.08 | 106 | 0.02 | 2950 |  | 0.8 | 0.01 | 0.003 | 0.01 | 0.04 |  | 3 | 0.001 | 1 |
|  |  |  | 0.1 |  | 94 |  |  | 76 |  |  |  | 5 | 0.2 | 0.04 | 138 | 0.02 | 3900 |  | 0.4 | 0.007 | 0.003 | 0.01 | 0.05 |  | 3 |  | 0.6 |
|  | **Mean** |  | 0.2 |  | 51 | 114 |  | 152 |  |  | 0.4 | 5 | 0.1 | 0.07 | 116 | 0.02 | 2590 |  | 0.8 | 0.01 | 0.003 | 0.01 | 0.04 |  | 3 | 0.001 | 0.7 |
|  | **Std dev** |  | 0.09 |  | 30 | 0 |  | 51 |  |  | 0.000 | 2 | 0.06 | 0.03 | 13 | 0.002 | 923 |  | 0.3 | 0.008 | 0.000 | 0.002 | 0.01 |  | 0.3 | 0.000 | 0.3 |
| **13193/6 Gollnitz,**  **SK** |  |  |  |  | 11 |  |  | 10 |  |  | 0.2 |  |  |  | 2410 |  | 49 |  | 0.005 | 0.006 | 0.003 | 0.02 |  |  | 0.03 |  | 0.001 |
|  |  |  | 0.02 |  | 12 |  |  | 9 |  |  | 0.2 | 1 |  |  | 2760 |  | 75 |  | 0.008 | 0.004 | 0.003 | 0.01 | 0.003 | 0.001 | 0.05 |  | 0.003 |
|  |  |  | 0.08 |  | 40 |  |  | 15 |  |  | 0.3 | 13 | 0.05 | 0.3 | 1410 | 0.02 | 166 |  | 0.03 | 0.004 | 0.004 | 0.02 | 0.01 |  | 0.2 |  | 0.03 |
|  |  |  | 0.06 |  | 26 |  |  | 16 |  |  | 0.2 | 8 | 0.04 | 0.2 | 2460 |  | 195 |  | 0.02 | 0.004 | 0.003 | 0.02 | 0.01 |  | 0.2 |  | 0.04 |
|  | **Mean** |  | 0.05 |  | 22 |  |  | 13 |  |  | 0.2 | 7 | 0.04 | 0.3 | 2260 | 0.02 | 121 |  | 0.02 | 0.005 | 0.003 | 0.01 | 0.009 | 0.001 | 0.1 |  | 0.02 |
|  | **Std dev** |  | 0.02 |  | 12 |  |  | 3 |  |  | 0.04 | 5 | 0.004 | 0.05 | 509 | 0.000 | 61 |  | 0.01 | 0.001 | 0.001 | 0.003 | 0.004 | 0.000 | 0.08 |  | 0.02 |
| **13228/41 Chessy,**  **FR** |  | 0.1 |  |  | 701 | 1133 |  | 18 | 0.2 | 0.2 | 0.4 | 40 | 0.03 |  | 12 | 0.2 |  |  | 0.1 | 0.3 | 0.005 | 0.05 | 0.01 | 0.01 | 112 | 0.1 | 0.001 |
|  |  | 0.10 |  |  | 1072 | 1302 | 0.8 | 18 | 4 | 1.0 | 0.7 | 630 |  | 0.04 | 17 | 0.3 | 0.1 |  | 0.1 | 0.4 | 0.008 | 1 | 0.01 | 0.02 | 199 | 0.1 | 0.01 |
|  |  | 0.1 |  |  | 598 | 700 | 0.5 | 20 | 0.4 | 0.2 | 0.2 | 41 |  |  | 19 | 0.2 | 0.05 |  | 0.2 | 0.3 | 0.005 | 0.06 | 0.006 | 0.01 | 597 | 0.1 | 0.005 |
|  |  | 0.04 |  |  | 116 | 150 |  | 13 |  | 0.04 | 0.1 | 7 |  |  | 65 | 0.1 |  |  | 0.03 | 0.5 | 0.003 | 0.02 |  | 0.02 | 88 | 0.4 | 0.002 |
|  | **Mean** | 0.09 |  |  | 622 | 821 | 0.6 | 17 | 2 | 0.4 | 0.4 | 179 | 0.03 | 0.04 | 28 | 0.2 | 0.09 |  | 0.1 | 0.4 | 0.005 | 0.4 | 0.01 | 0.01 | 249 | 0.2 | 0.005 |
|  | **Std dev** | 0.03 |  |  | 341 | 445 | 0.1 | 2 | 2 | 0.4 | 0.2 | 260 | 0.000 | 0.000 | 21 | 0.09 | 0.03 |  | 0.05 | 0.07 | 0.002 | 0.6 | 0.003 | 0.004 | 205 | 0.1 | 0.004 |
| **13247/60 Solotuschinsk,**  **RU** |  | 0.2 |  |  | 1677 | 1840 | 0.6 |  |  |  |  | 37 |  | 0.08 | 425 |  | 0.3 |  | 0.03 | 0.9 | 0.003 | 0.02 | 0.03 | 0.008 | 42 | 0.001 | 0.01 |
|  |  | 0.05 |  |  | 470 | 477 |  | 9 |  |  | 0.2 | 10 |  |  | 879 |  |  |  | 0.01 | 0.2 | 0.003 | 0.01 | 0.005 |  | 13 | 0.001 | 0.005 |
|  |  |  |  |  | 88 |  |  |  |  |  |  | 2 |  |  | 31 |  | 0.04 |  | 0.004 | 0.06 | 0.003 |  |  | 0.001 | 15 | 0.001 | 0.001 |
|  |  | 0.1 |  |  | 1190 | 1460 | 0.8 | 14 |  |  | 0.2 | 32 | 0.02 |  | 1235 | 0.02 | 2 | 0.02 | 0.01 | 0.01 | 0.003 | 0.01 |  |  | 65 | 0.007 | 0.7 |
|  | **Mean** | 0.1 |  |  | 856 | 1259 | 0.7 | 11 |  |  | 0.2 | 20 | 0.02 | 0.08 | 642 | 0.02 | 0.7 | 0.02 | 0.02 | 0.3 | 0.003 | 0.01 | 0.02 | 0.004 | 34 | 0.002 | 0.2 |
|  | **Std dev** | 0.05 |  |  | 617 | 574 | 0.09 | 2 |  |  | 0.04 | 14 | 0.000 | 0.000 | 455 | 0.000 | 0.8 | 0.000 | 0.01 | 0.4 | 0.000 | 0.003 | 0.01 | 0.004 | 21 | 0.003 | 0.3 |
| **13240/53 Cornwall,**  **UK** |  |  | 0.02 |  | 92 | 130 | 22 | 8 | 1 | 0.1 | 0.5 | 105 |  |  | 48 | 0.02 | 0.6 | 0.2 | 0.07 | 2 | 0.008 | 0.01 | 0.1 | 0.02 | 26 | 0.01 | 0.02 |
|  |  | 0.06 | 0.02 |  | 77 | 210 | 23 |  | 5 | 0.3 | 1 | 440 |  |  | 315 |  | 1 | 0.3 | 0.06 | 1.0 | 0.01 | 0.02 | 0.1 | 0.02 | 15 | 0.02 | 0.05 |
|  | **Mean** | 0.06 | 0.02 |  | 85 | 170 | 23 | 8 | 3 | 0.2 | 0.9 | 273 |  |  | 182 | 0.02 | 0.9 | 0.3 | 0.06 | 2 | 0.01 | 0.02 | 0.1 | 0.02 | 20 | 0.02 | 0.03 |
|  | **Std dev** | 0.000 | 0.002 |  | 8 | 40 | 0.8 | 0.000 | 2 | 0.09 | 0.4 | 168 |  |  | 133 | 0.000 | 0.3 | 0.04 | 0.004 | 0.5 | 0.004 | 0.003 | 0.005 | 0.001 | 6 | 0.003 | 0.01 |
| **13238/51 Chessy,**  **FR** |  | 0.2 | 0.1 |  | 309 | 1140 | 10 | 16 | 1.0 | 3 | 0.1 | 62 | 0.05 | 0.08 | 2 | 0.8 | 17 | 0.1 | 0.4 | 0.1 | 0.005 | 244 | 5 | 0.02 | 11 | 0.005 | 5 |
|  |  |  | 0.02 | 4 | 91 | 297 | 2 | 12 | 0.2 | 0.6 |  | 52 |  |  | 0.2 | 0.1 | 3 |  | 0.05 | 0.04 | 0.003 | 41 | 0.6 | 0.001 | 8 | 0.001 | 0.5 |
|  | **Mean** | 0.2 | 0.06 | 4 | 200 | 719 | 6 | 14 | 0.6 | 2 | 0.1 | 57 | 0.05 | 0.08 | 1.0 | 0.5 | 10 | 0.1 | 0.2 | 0.08 | 0.004 | 142 | 3 | 0.01 | 9 | 0.003 | 3 |
|  | **Std dev** | 0 | 0.04 | 0 | 109 | 422 | 4 | 2 | 0.4 | 1 | 0 | 5 | 0 | 0 | 0.8 | 0.3 | 7 | 0 | 0.2 | 0.04 | 0.0008 | 102 | 2 | 0.01 | 2 | 0.002 | 2 |

**Table S3**. LA-ICP-MS data (ppm) for REEs in all the analysed points of the azurite samples (Countries abbreviations as in Table 2), with LOD calculated according to [24]

| **Sample** |  | **La** | **Ce** | **Pr** | **Nd** | **Sm** | **Eu** | **Gd** | **Tb** | **Dy** | **Ho** | **Er** | **Tm** | **Yb** | **Lu** | **Y** |
| --- | --- | --- | --- | --- | --- | --- | --- | --- | --- | --- | --- | --- | --- | --- | --- | --- |
| **LOD [24]** |  | 0.0005 | 0.0004 | 0.0009 | 0.002 | 0.007 | 0.0008 | 0.002 | 0.0004 | 0.002 | 0.0004 | 0.001 | 0.001 | 0.002 | 0.0003 | 0.002 |
| **13202/15 Wolwodina,**  **RO** |  | 18 | 41 | 9 | 41 | 9 | 4 | 7 | 1 | 7 | 1 | 4 | 0.7 | 5 | 0.7 | 31 |
|  |  | 28 | 61 | 14 | 66 | 15 | 7 | 12 | 2 | 11 | 2 | 6 | 0.9 | 6 | 0.9 | 62 |
|  |  | 35 | 77 | 18 | 86 | 21 | 10 | 17 | 3 | 17 | 3 | 9 | 1 | 9 | 1 | 90 |
|  |  | 42 | 89 | 18 | 81 | 19 | 9 | 15 | 2 | 15 | 3 | 9 | 1 | 9 | 1 | 74 |
|  | **Average** | 31 | 67 | 15 | 68 | 16 | 8 | 13 | 2 | 12 | 3 | 7 | 1 | 7 | 1 | 64 |
|  | **Std dev** | 9 | 18 | 4 | 18 | 5 | 2 | 4 | 0.7 | 4 | 0.8 | 2 | 0.3 | 2 | 0.2 | 22 |
| **132107/23 Siegen,**  **GE** |  | 1 | 3 | 0.4 | 2 | 0.8 | 0.3 | 2 | 0.6 | 6 | 2 | 6 | 1 | 8 | 1 | 49 |
|  |  | 12 | 20 | 3 | 10 | 2 | 0.6 | 3 | 0.7 | 6 | 1 | 5 | 0.7 | 5 | 0.8 | 45 |
|  |  | 5 | 11 | 2 | 7 | 2 | 0.4 | 2 | 0.3 | 2 | 0.7 | 2 | 0.4 | 3 | 0.4 | 18 |
|  |  | 2 | 5 | 0.9 | 4 | 1 | 0.2 | 0.9 | 0.1 | 1.0 | 0.3 | 1 | 0.3 | 3 | 0.5 | 4 |
|  | **Average** | 5 | 10 | 1 | 6 | 2 | 0.4 | 2 | 0.4 | 4 | 1 | 4 | 0.6 | 5 | 0.7 | 29 |
|  | **Std dev** | 4 | 6 | 0.9 | 3 | 0.6 | 0.1 | 0.7 | 0.2 | 2 | 0.6 | 2 | 0.3 | 2 | 0.3 | 19 |
| **13216/29 Chessy,**  **FR** |  | 0.6 | 0.7 | 0.2 | 1 | 0.4 | 0.2 | 0.6 | 0.2 | 2 | 0.6 | 2 | 0.4 | 3 | 0.5 | 9 |
|  |  | 0.6 | 0.9 | 0.2 | 0.9 | 0.2 | 0.1 | 0.3 | 0.07 | 0.6 | 0.2 | 0.6 | 0.08 | 0.6 | 0.10 | 3 |
|  |  | 0.7 | 1.0 | 0.2 | 1 | 0.3 | 0.2 | 0.3 | 0.07 | 0.5 | 0.1 | 0.5 | 0.08 | 0.6 | 0.09 | 3 |
|  |  | 0.4 | 0.6 | 0.1 | 0.6 | 0.2 | 0.09 | 0.2 | 0.04 | 0.3 | 0.09 | 0.3 | 0.05 | 0.4 | 0.06 | 2 |
|  | **Average** | 0.6 | 0.8 | 0.2 | 0.9 | 0.3 | 0.2 | 0.4 | 0.09 | 0.8 | 0.3 | 0.9 | 0.2 | 1 | 0.2 | 4 |
|  | **Std dev** | 0.09 | 0.1 | 0.03 | 0.2 | 0.08 | 0.06 | 0.1 | 0.06 | 0.6 | 0.2 | 0.8 | 0.1 | 1 | 0.2 | 3 |
| **13466/79bis Campiglia,**  **IT** |  | 0.01 | 0.04 | 0.009 | 0.04 | 0.02 | 0.006 | 0.01 | 0.003 | 0.03 | 0.007 | 0.03 | 0.01 | 0.1 | 0.02 | 0.07 |
|  |  | 0.02 | 0.1 | 0.03 | 0.2 | 0.2 | 0.08 | 0.3 | 0.09 | 0.8 | 0.2 | 1 | 0.4 | 5 | 1 | 3 |
|  |  | 0.002 | 0.007 | 0.002 | 0.01 |  | 0.001 | 0.003 | 0.001 | 0.004 | 0.001 | 0.005 | 0.001 | 0.01 | 0.002 | 0.02 |
|  |  | 0.003 | 0.01 | 0.004 | 0.03 | 0.009 | 0.006 | 0.01 | 0.002 | 0.01 | 0.001 | 0.004 |  |  | 0.000 | 0.02 |
|  | **Average** | 0.009 | 0.04 | 0.01 | 0.08 | 0.07 | 0.02 | 0.08 | 0.02 | 0.2 | 0.06 | 0.3 | 0.1 | 2 | 0.3 | 0.7 |
|  | **Std dev** | 0.007 | 0.04 | 0.01 | 0.09 | 0.09 | 0.03 | 0.1 | 0.04 | 0.3 | 0.10 | 0.5 | 0.2 | 3 | 0.6 | 1 |
| **14890/83 Wolwodina,**  **RO** |  | 0.03 | 0.03 | 0.005 | 0.02 |  | 0.003 | 0.02 | 0.002 | 0.01 | 0.003 | 0.01 | 0.001 | 0.01 | 0.002 | 0.4 |
|  |  | 0.02 | 0.01 | 0.003 | 0.02 | 0.01 | 0.002 | 0.01 | 0.002 | 0.009 | 0.003 | 0.009 | 0.002 | 0.006 | 0.002 | 0.4 |
|  |  | 0.01 | 0.01 | 0.003 | 0.02 | 0.008 | 0.001 | 0.005 | 0.001 | 0.006 | 0.001 | 0.003 |  |  | 0.000 | 0.2 |
|  |  | 0.02 | 0.008 | 0.002 | 0.01 |  | 0.003 | 0.007 | 0.001 | 0.007 | 0.002 | 0.003 |  | 0.003 |  | 0.2 |
|  | **Average** | 0.02 | 0.02 | 0.003 | 0.02 | 0.01 | 0.002 | 0.01 | 0.001 | 0.008 | 0.002 | 0.007 | 0.001 | 0.007 | 0.001 | 0.3 |
|  | **Std dev** | 0.006 | 0.007 | 0.001 | 0.004 | 0.002 | 0.001 | 0.004 | 0.000 | 0.002 | 0.001 | 0.004 | 0.000 | 0.003 | 0.001 | 0.1 |
| **16695/86 Laurion,**  **GR** |  | 0.01 | 0.02 | 0.005 | 0.02 | 0.009 | 0.001 | 0.004 | 0.001 | 0.003 | 0.001 | 0.003 |  | 0.006 | 0.001 | 0.02 |
|  |  | 0.09 | 1 | 0.03 | 0.1 | 0.04 | 0.01 | 0.06 | 0.02 | 0.2 | 0.05 | 0.2 | 0.04 | 0.5 | 0.10 | 0.8 |
|  |  | 0.1 | 0.3 | 0.05 | 0.2 | 0.06 | 0.02 | 0.07 | 0.01 | 0.09 | 0.02 | 0.09 | 0.01 | 0.1 | 0.01 | 0.4 |
|  |  | 0.05 | 1 | 0.03 | 0.2 | 0.08 | 0.03 | 0.1 | 0.04 | 0.4 | 0.2 | 0.7 | 0.2 | 2 | 0.3 | 3 |
|  | **Average** | 0.07 | 0.7 | 0.03 | 0.1 | 0.05 | 0.01 | 0.06 | 0.02 | 0.2 | 0.06 | 0.2 | 0.09 | 0.6 | 0.09 | 1.0 |
|  | **Std dev** | 0.04 | 0.6 | 0.01 | 0.06 | 0.03 | 0.009 | 0.04 | 0.01 | 0.2 | 0.06 | 0.3 | 0.09 | 0.8 | 0.1 | 1 |
| **16696/87 Laurion,**  **GR** |  | 0.08 | 0.004 | 0.02 | 0.1 | 0.04 | 0.007 | 0.1 | 0.02 | 0.1 | 0.04 | 0.1 | 0.03 | 0.2 | 0.04 | 2 |
|  |  | 0.06 | 0.003 | 0.01 | 0.09 | 0.05 | 0.009 | 0.1 | 0.03 | 0.3 | 0.1 | 0.5 | 0.1 | 1 | 0.2 | 5 |
|  |  | 0.06 | 0.006 | 0.02 | 0.09 | 0.05 | 0.006 | 0.08 | 0.01 | 0.09 | 0.03 | 0.1 | 0.02 | 0.1 | 0.03 | 2 |
|  |  | 0.05 | 0.005 | 0.01 | 0.06 | 0.03 | 0.005 | 0.07 | 0.02 | 0.1 | 0.03 | 0.1 | 0.02 | 0.1 | 0.03 | 2 |
|  | **Average** | 0.06 | 0.004 | 0.02 | 0.09 | 0.04 | 0.007 | 0.1 | 0.02 | 0.2 | 0.05 | 0.2 | 0.04 | 0.4 | 0.08 | 3 |
|  | **Std dev** | 0.01 | 0.001 | 0.003 | 0.02 | 0.006 | 0.002 | 0.03 | 0.008 | 0.09 | 0.04 | 0.2 | 0.04 | 0.4 | 0.09 | 1 |
| **13193/6 Gollnitz,**  **SK** |  | 0.2 | 0.3 | 0.07 | 0.4 | 0.08 | 0.04 | 0.1 | 0.05 | 0.5 | 0.2 | 1 | 0.3 | 3 | 0.5 | 5 |
|  |  | 0.5 | 1 | 0.2 | 1 | 0.3 | 0.1 | 0.3 | 0.06 | 0.8 | 0.4 | 2 | 0.5 | 4 | 0.6 | 7 |
|  |  | 2 | 3 | 0.3 | 1 | 0.2 | 0.06 | 0.1 | 0.03 | 0.3 | 0.09 | 0.4 | 0.08 | 0.8 | 0.1 | 3 |
|  |  | 1 | 2 | 0.3 | 0.9 | 0.1 | 0.05 | 0.1 | 0.03 | 0.2 | 0.07 | 0.3 | 0.06 | 0.6 | 0.1 | 2 |
|  | **Average** | 0.9 | 1 | 0.2 | 0.9 | 0.2 | 0.06 | 0.2 | 0.04 | 0.5 | 0.2 | 1.0 | 0.2 | 2 | 0.3 | 4 |
|  | **Std dev** | 0.6 | 0.9 | 0.09 | 0.3 | 0.06 | 0.02 | 0.06 | 0.01 | 0.2 | 0.1 | 0.7 | 0.2 | 1 | 0.2 | 2 |
| **13228/41 Chessy,**  **FR** |  | 0.003 | 0.005 | 0.005 | 0.03 | 0.03 | 0.004 | 0.06 | 0.03 | 0.5 | 0.2 | 1 | 0.2 | 2 | 0.3 | 3 |
|  |  | 0.002 | 0.01 | 0.007 | 0.05 | 0.05 | 0.008 | 0.09 | 0.04 | 0.6 | 0.2 | 1 | 0.2 | 1 | 0.2 | 4 |
|  |  | 0.006 | 0.01 | 0.02 | 0.1 | 0.09 | 0.02 | 0.2 | 0.06 | 0.8 | 0.3 | 1 | 0.2 | 1 | 0.2 | 5 |
|  |  | 0.001 | 0.003 | 0.004 | 0.03 | 0.02 | 0.002 | 0.04 | 0.03 | 0.5 | 0.3 | 1 | 0.3 | 3 | 0.5 | 4 |
|  | **Average** | 0.1 | 0.04 | 0.1 | 0.9 | 0.4 | 0.1 | 0.3 | 0.05 | 0.4 | 0.1 | 0.6 | 0.2 | 3 | 0.7 | 0.9 |
|  | **Std dev** | 0.002 | 0.004 | 0.005 | 0.04 | 0.03 | 0.005 | 0.05 | 0.01 | 0.1 | 0.03 | 0.1 | 0.04 | 0.5 | 0.1 | 0.8 |
| **13247/60 Solotuschinsk,**  **RU** |  | 0.03 | 0.04 | 0.01 | 0.08 | 0.04 | 0.02 | 0.04 | 0.009 | 0.09 | 0.05 | 0.5 | 0.3 | 4 | 1.0 | 0.3 |
|  |  | 0.02 | 0.01 | 0.007 | 0.04 | 0.02 | 0.008 | 0.02 | 0.003 | 0.03 | 0.01 | 0.03 | 0.006 | 0.09 | 0.03 | 0.1 |
|  |  | 0.007 | 0.02 | 0.005 | 0.03 | 0.01 | 0.009 | 0.02 | 0.006 | 0.06 | 0.04 | 0.4 | 0.2 | 3 | 0.9 | 0.2 |
|  |  | 0.5 | 0.1 | 0.6 | 3 | 1 | 0.5 | 1 | 0.2 | 1 | 0.4 | 2 | 0.4 | 4 | 0.7 | 3 |
|  | **Average** | 0.1 | 0.04 | 0.1 | 0.9 | 0.4 | 0.1 | 0.3 | 0.05 | 0.4 | 0.1 | 0.6 | 0.2 | 3 | 0.7 | 0.9 |
|  | **Std dev** | 0.2 | 0.04 | 0.2 | 1 | 0.6 | 0.2 | 0.5 | 0.08 | 0.5 | 0.1 | 0.6 | 0.1 | 2 | 0.4 | 1 |
| **13240/53 Cornwall,**  **UK** |  | 0.03 | 0.09 | 0.02 | 0.08 | 0.03 | 0.01 | 0.03 | 0.006 | 0.04 | 0.01 | 0.06 | 0.02 | 0.3 | 0.07 | 0.1 |
|  |  | 0.009 | 0.06 | 0.01 | 0.05 | 0.03 | 0.006 | 0.02 | 0.005 | 0.02 | 0.007 | 0.03 | 0.005 | 0.08 | 0.01 | 0.08 |
|  | **Average** | 0.02 | 0.08 | 0.01 | 0.06 | 0.03 | 0.01 | 0.02 | 0.006 | 0.03 | 0.01 | 0.05 | 0.01 | 0.2 | 0.04 | 0.1 |
|  | **Std dev** | 0.01 | 0.01 | 0.002 | 0.02 | 0.002 | 0.004 | 0.007 | 0.001 | 0.01 | 0.003 | 0.02 | 0.007 | 0.09 | 0.03 | 0.02 |
| **13238/51 Chessy,**  **FR** |  | 0.7 | 2 | 1 | 10 | 8 | 2 | 10 | 3 | 21 | 5 | 16 | 3 | 22 | 3 | 116 |
|  |  | 0.2 | 0.7 | 0.4 | 4 | 3 | 0.7 | 2 | 0.3 | 1 | 0.2 | 0.6 | 0.1 | 1 | 0.2 | 5 |
|  | **Average** | 0.5 | 2 | 0.8 | 7 | 5 | 1 | 6 | 1 | 11 | 3 | 9 | 2 | 11 | 2 | 61 |
|  | **Std dev** | 0.3 | 0.9 | 0.4 | 3 | 2 | 0.7 | 4 | 1 | 10 | 2 | 8 | 1 | 10 | 1 | 56 |


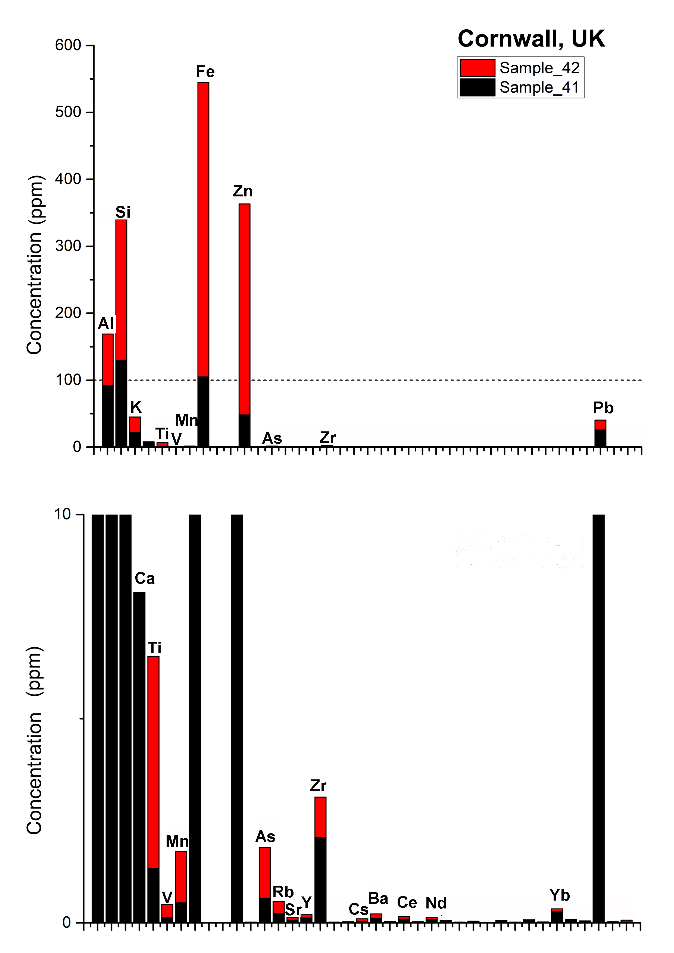


**Fig. S4**. Histogram showing the concentration of all elements detected in the sample from Cornwall, UK; the different colours correspond to different points of LA-ICP-MS analysis
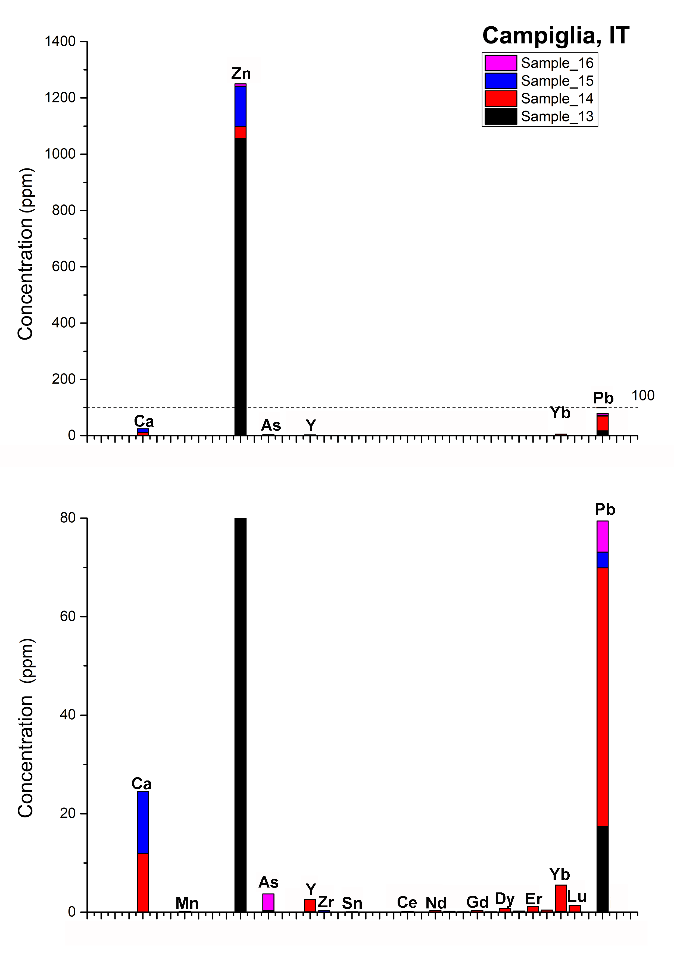


**Fig. S5**. Histogram showing the concentration of all elements detected in the sample from Campiglia, Italy; the different colours correspond to different points of LA-ICP-MS analysis


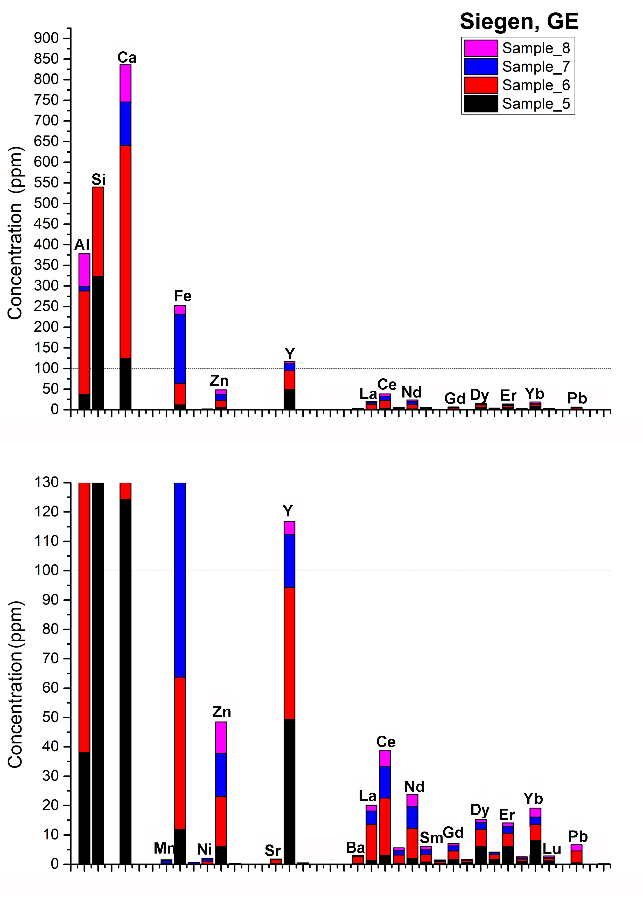


**Fig. S6**. Histogram showing the concentration of all elements detected in the sample from Siegen, Germany; the different colours correspond to different points of LA-ICP-MS analysis


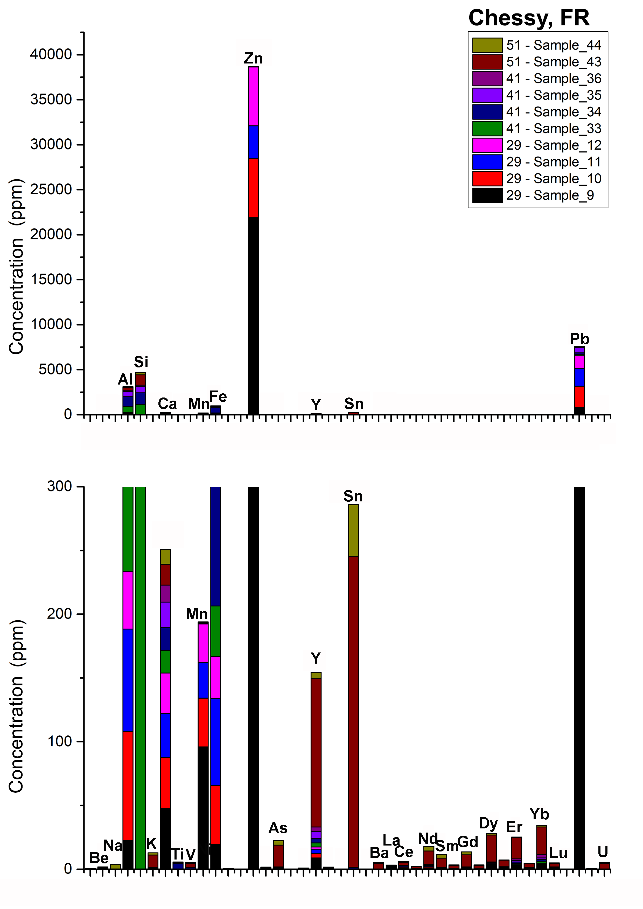


**Fig. S7**. Histogram showing the concentration of all elements detected in the samples from Chessy, France; the different colours correspond to different points of LA-ICP-MS analysis, the different sample is indicated by the first 2 digits in the legend (corresponding to the last 2 digits of the MUST code)


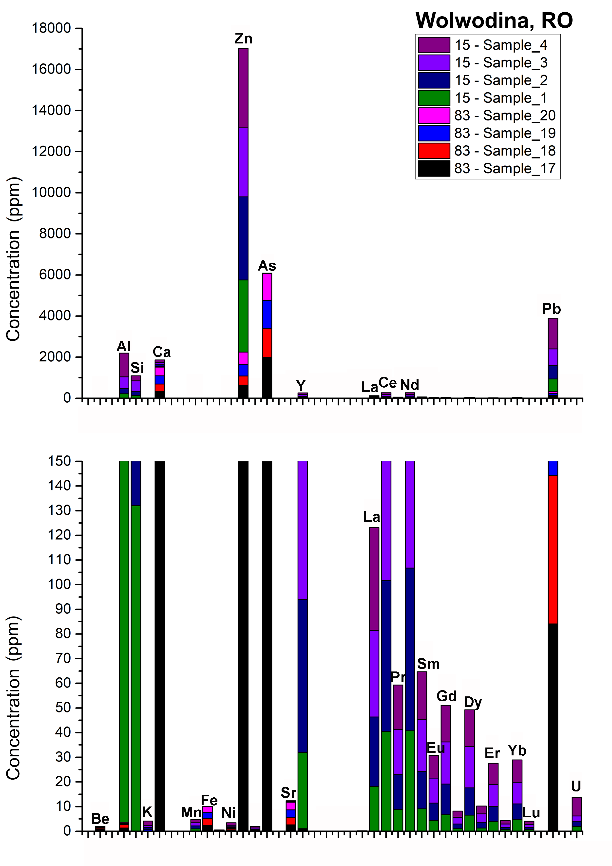


**Fig. S8**. Histogram showing the concentration of all elements detected in the samples from Wolwodina, Romania; the different colours correspond to different points of LA-ICP-MS analysis, the different sample is indicated by the first 2 digits in the legend (corresponding to the last 2 digits of the MUST code)


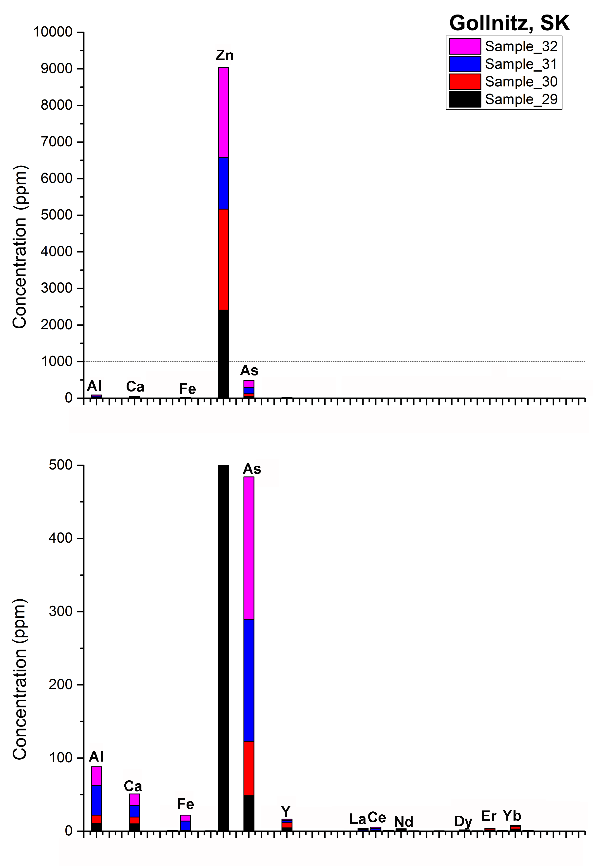


**Fig. S9**. Histogram showing the concentration of all elements detected in the sample from Gollnitz, Slovakia; the different colours correspond to different points of LA-ICP-MS analysis


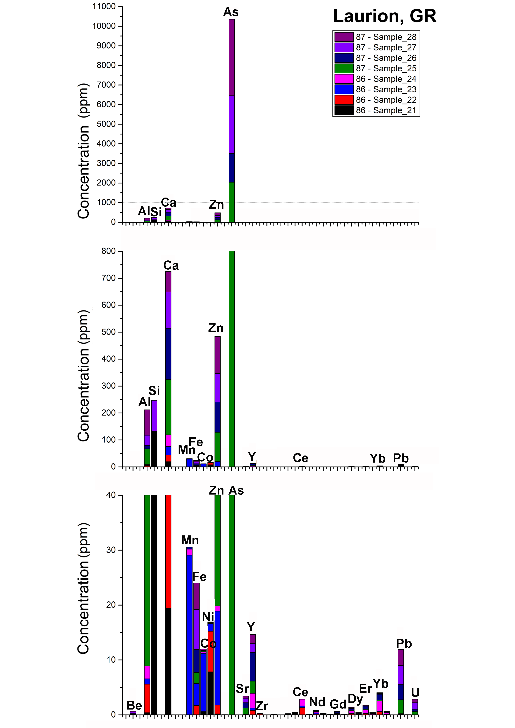


**Fig. S10**. Histogram showing the concentration of all elements detected in the samples from Laurion, Greece; the different colours correspond to different points of LA-ICP-MS analysis, the different sample is indicated by the first 2 digits in the legend (corresponding to the last 2 digits of the MUST code)


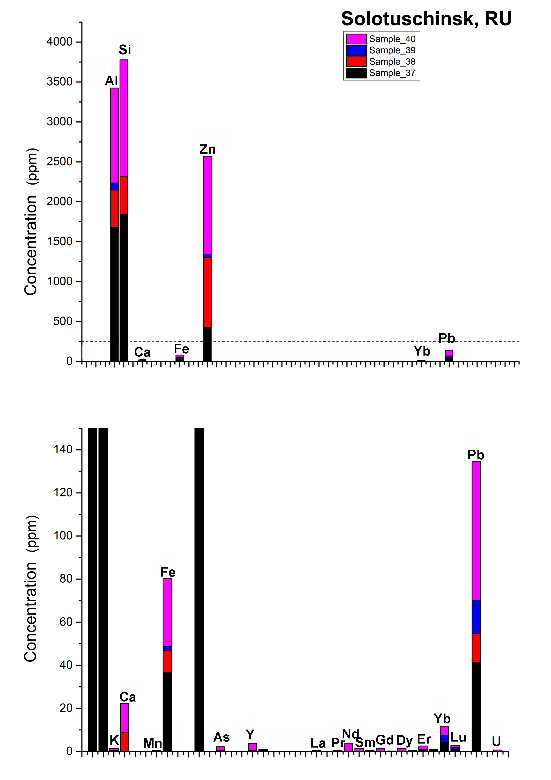


**Fig. S11**. histogram showing the concentration of all elements detected in the sample from Solotuschinsk, Russia; the different colours correspond to different points of LA-ICP-MS analysis
